# Identification of CFHR4 as a Potential Prognosis Biomarker Associated With lmmune Infiltrates in Hepatocellular Carcinoma

**DOI:** 10.3389/fimmu.2022.892750

**Published:** 2022-06-22

**Authors:** Hongjun Yu, Chaoqun Wang, Shanjia Ke, Miaoyu Bai, Yanan Xu, Shounan Lu, Zhigang Feng, Baolin Qian, Yue Xu, Menghua Zhou, Zihao Li, Bing Yin, Xinglong Li, Yongliang Hua, Yongzhi Zhou, Shangha Pan, Yao Fu, Yong Ma

**Affiliations:** ^1^Department of Minimal Invasive Hepatic Surgery, The First Affiliated Hospital of Harbin Medical University, Harbin, China; ^2^Key Laboratory of Hepatosplenic Surgery, Ministry of Education, The First Affiliated Hospital of Harbin Medical University, Harbin, China; ^3^The First Department of General Surgery, Affiliated Hospital of Inner Mongolia Minzu University, Tongliao, China; ^4^Department of Pediatrics, Hainan Hospital of PLA General Hospital, Sanya, China; ^5^Department of Pediatric Surgery, The First Affiliated Hospital of Harbin Medical University, Harbin, China; ^6^Department of Ultrasound, The First Affiliated Hospital of Harbin Medical University, Harbin, China

**Keywords:** CFHR4, prognosis, biomarker, immune Infiltrate, hepatocellular carcinoma

## Abstract

**Background:**

Complement factor H-related 4 (CFHR4) is a protein-coding gene that plays an essential role in multiple diseases. However, the prognostic value of CFHR4 in hepatocellular carcinoma (HCC) is unknown.

**Methods:**

Using multiple databases, we investigated CFHR4 expression levels in HCC and multiple cancers. The relationship between CFHR4 expression levels and clinicopathological variables was further analyzed. Various potential biological functions and regulatory pathways of CFHR4 in HCC were identified by performing a Gene Ontology (GO) analysis, Kyoto Encyclopedia of Genes and Genomes (KEGG) analysis and Gene Set Enrichment Analysis (GSEA). Single-sample gene set enrichment analysis (ssGSEA) was performed to confirm the correlation between CFHR4 expression and immune cell infiltration. The correlations between CFHR4 expression levels in HCC and N6-methyladenosine (m6A) modifications and the competing endogenous RNA (ceRNA) regulatory networks were confirmed in TCGA cohort.

**Results:**

CFHR4 expression levels were significantly decreased in HCC tissues. Low CFHR4 expression in HCC tissues was significantly correlated with the patients’ sex, race, age, TNM stage, pathological stage, tumor status, residual tumor, histologic grade and alpha fetal protein (AFP) level. GO and KEGG analyses revealed that differentially expressed genes related to CFHR4 may be involved in the synaptic membrane, transmembrane transporter complex, gated channel activity, chemical carcinogenesis, retinol metabolism, calcium signaling pathway, PPAR signaling pathway, insulin and gastric acid secretion. GSEA revealed that the FCGR-activated reaction, PLK1 pathway, ATR pathway, MCM pathway, cascade reactions of PI3K and FGFR1, reactant-mediated MAPK activation and FOXM1 pathway were significantly enriched in HCC with low CFHR4 expression. Moreover, CFHR4 expression was inversely correlated the levels of infiltrating Th2 cells, NK CD56bright cells and Tfh cells. In contrast, we observed positive correlations with the levels of infiltrating DCs, neutrophils, Th17 cells and mast cells. CFHR4 expression showed a strong correlation with various immunomarker groups in HCC. In addition, high CFHR4 expression significantly prolonged the overall survival (OS), disease-specific survival (DSS) and progression-free interval (PFI). We observed a substantial correlation between the expression of CFHR4 and multiple N6-methyladenosine genes in HCC and constructed potential CFHR4-related ceRNA regulatory networks.

**Conclusions:**

CFHR4 might be a potential therapeutic target for improving the HCC prognosis and is closely related to immune cell infiltration.

## Introduction

HCC is the sixth most common cancer worldwide. Over 900,000 new cases of HCC are confirmed each year, and approximately 800,000 people die of HCC annually, making it the third most common cause of cancer-related death. The morbidity and mortality rates of HCC are 2 to 3 times higher in men than in women in most areas (1). In China, the death rate of HCC is the highest among men over 60 years of age. The number of new cases of liver cancer diagnosed each year accounts for approximately 50% of all cases worldwide. The key determinants of liver cancer are chronic HBV infection, aflatoxin exposure or both (1, 2). The development of surgical procedures has improved the survival rate of patients with early-phase HCC, but many patients already have advanced HCC at the diagnosis, resulting in a poor overall survival rate. Therefore, the identification of new, relevant biomarkers is urgently needed to improve the early diagnosis, prognostic assessment and treatment of HCC (3–5).

Research shows that the complement system is a vitally important component of innate immunity and is extensively involved in innate immune recognition, adaptive cell stimulation and proinflammatory effector responses. The complement system exerts a regulatory effect on the tumor microenvironment, influencing the outcome of the immune response (6, 7). The factor H/CFHR family includes five complement F factor H-related proteins (CFHR1/2/3/4/5), factor H and complement factor H‐like protein (CFHL1) (8, 9). CFHRs are secreted plasma proteins synthesized mainly by hepatocytes. CFHR4 is a key component of the innate immune system, and its expression is restricted to the liver (10). To date, numerous studies have suggested a role for CFHR4 in immune system disorders, such as age-related macular degeneration (AMD) (10, 11), systemic lupus erythematosus (12) and atypical hemolytic uremic syndrome (AHUS) (13, 14). However, the association of CFHR4 with HCC has not yet been characterized.

The N6-methyladenosine (m6A) RNA and competing endogenous RNA (ceRNA) regulatory network is currently a new direction in cancer therapy, and the mechanisms have been extensively studied in HCC (15). Current studies mainly focus on methyltransferases, demethylases and binding proteins (16, 17). Although the mechanism of the m6A regulatory factor requires further study, the roles of the m6A regulatory factor in tumor proliferation, invasion and metastasis have been confirmed (18). In addition, ceRNA regulatory networks are also crucial for the emergence and development of multiple cancers, including ovarian cancer (19), esophageal cancer (20) and gastric cancer (21). However, no studies have examined the ceRNA regulatory network of CFHR4 in HCC or reported on its association with m6A regulators.

In the present study, we analyzed CFHR4 expression levels in HCC tumors and normal liver tissue from multiple datasets. An analysis of RNA sequencing (RNA-seq) data from TCGA revealed the clinical relevance and potential diagnostic and prognostic roles of CFHR4 in HCC. In addition, we further explored the biological significance of CFHR4 by performing enrichment analyses and a protein–protein interaction (PPI) network analysis and determining the correlation with immune cell infiltration. After analyzing the correlation of CFHR4 and m6A, we constructed ceRNA regulatory networks involving CFHR4 in HCC.

## Materials and Methods

### RNA-Seq Data Source

We first collected gene expression data and clinical data from 424 patients with HCC in TCGA (https://portal.gdc.cancer.gov). In addition, the RNA sequencing data (GSE14520) were downloaded from the Gene Expression Omnibus (GEO) database. HTSeq-FPKM of level 3 format was converted into transcripts per million (TPM). Screening was performed to exclude patients with incomplete information, and the TPM data from 374 patients were used in subsequent analyses (**Supplementary Table 1**). The evolution process used the “ggplot2” R package.

### Cell Lines and Cell Culture

Normal human liver cells (WRL68) were purchased from AcceGen (Fairfield, USA), and HCC cell lines (BEL7402, SK-hep1, HCCLM3, HepG2 and Huh7) were purchased from the Chinese Academy of Science (Shanghai, China). WRL68 cells were cultured in RPMI-1640 medium, and other cell lines were cultured in DMEM supplemented with 10% FBS and 1% penicillin-streptomycin. All cells were incubated in a 37°C incubator with 5% CO_2_.

### HCC Tissue Collection

We collected 30 pairs of HCC tissues and adjacent liver tissues at the First Affiliated Hospital of Harbin Medical University from 2006 to 2013 after obtaining informed consent from patients. The research project was conducted under the supervision of the Ethics Committee of the First Affiliated Hospital of Harbin Medical University.

### Quantitative Real-Time PCR

Quantitative real-time PCR was performed on the samples as described previously (5). The following primers were used: CFHR4-F, 5’-TGCGGTTTAAGCTCCATGACA -3’; CFHR4-R, 5’-CCCATCTTCACCACACACTATG-3’; GAPDH-F, 5’ -TGACTTCAACAGCGACACCCA-3’ and GAPDH-R, 5’-CACCCTGTTGCTGTAGCCAAA-3’. GAPDH was used as a control to determine changes in mRNA levels using the 2^-ΔΔCT^ method.

### Identification of Differentially Expressed Genes

The differentially expressed genes (DEGs) between high CFHR4 expression and low CFHR4 expression samples from TCGA database were analyzed using the DEseq2 (1.26.0) R package (22) with Student’s t test. Differences were considered statistically significant for an adjusted p value < 0.05 and absolute log2-fold change > 1.5. Moreover, volcano plots and heatmaps were constructed to visualize the DEGs.

### Gene Set Enrichment Analysis (GSEA)

Pathway enrichment analyses were performed with the “clusterProfiler” R package (23, 24). The c2.cp.v7.2.symbols.gmt curated gene sets were retrieved from the Molecular Signatures Database (MSigDB). Each analytical technique was conducted repeatedly a thousand times. An FDR-corrected q value < 0.25 and adjusted p value< 0.05 were considered statistically significant.

### ssGSEA of Immune Cell Infiltration

We analyzed the levels of infiltration of 24 types of immune cells in HCC using the ssGSEA method with the GSVA package in R. We then quantified the enrichment score for each immune cell by performing gene expression profiling of each HCC sample based on the signature of immune cells (25, 26).

### Construction and Evaluation of the Nomogram

The univariate Cox regression analysis of the correlation between CFHR4 expression and the values multiple clinical prognostic parameters in patients with HCC was performed using R software with the “survival” package. Using the RMS package (version 6.2-0) and survival package (version 3.2-10), nomograms including important clinical features and calibration plots were constructed. The 45° line represents the best-predicted value, and calibration curves were graphically evaluated by mapping the nomogram-predicted probability against observed occurrences. The consistency index (C-index) was used to measure the discriminative capability of the nomogram and to compare the predictive accuracy of nomograms and individual prognostic indicators. This process was calculated using the bootstrap method and repeated 1000 times. In the present study, one-way analysis of variance (ANOVA) and two-tailed Student’s t test were used to analyze the data. A P value < 0.05 was considered statistically significant.

### Prediction and Construction of ceRNA Networks

The TargetScan (http://www.targetscan.org), DIANA-microT (http://diana.imis.athena-innovation.gr/DianaTools/index) and RNAinter (http://www.rnainter.org) online sites were used together to predict and analyze the target miRNAs of CFHR4, compare the correlations between the expression of CFHR4 and target miRNAs and screen miRNAs that were more compatible with ceRNA networks. The target lncRNAs of the screened miRNAs were predicted and analyzed using miRNet2.0 (www.mirnet.ca/miRNet/home.xhtml) and starBase3.0 (www.starbase.sysu.edu.cn), and the correlation between the two was further analyzed to screen for additional eligible ceRNAs. A comprehensive analysis of negatively correlated miRNA–mRNA and miRNA-lncRNA expression levels was performed to establish an HCC-related lncRNA-miRNA–mRNA (CFHR4) ceRNA network.

### Statistical Analysis

The R package (version 3.6.3) was used for statistical analyses and plotting. CFHR4 expression in unpaired and paired samples was analyzed using the Wilcoxon rank sum test and Wilcoxon signed rank test, respectively, with the pROC (1.17.0.1) package for ROC analysis. In addition, the Kruskal–Wallis test and univariate Cox analysis were applied to investigate whether CFHR4 expression was associated with clinicopathological factors. Using the KM method and log-rank test, we compared the differences in 10-year OS, DSS and PFI between patients with high CFHR4 expression and those with low CFHR4 expression in TCGA. In all studies, a P value < 0.05 was defined as statistically significant.

## Results

### CFHR4 Expression Is Downregulated in HCC

**Figure 1:**
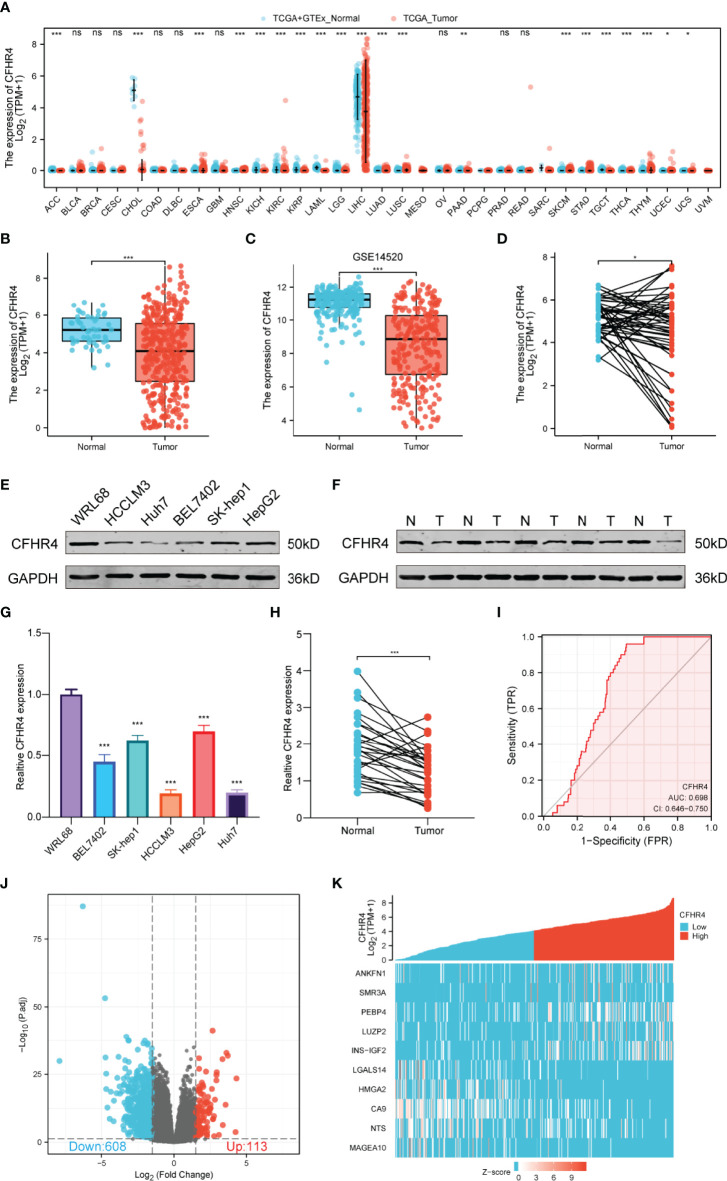
Differences in the expression of CFHR4 and CFHR4-associated DEGs. **(A)** CFHR4 expression levels in different cancer tissues compared to normal tissues (TCGA). **(B–D)** CFHR4 expression in HCC samples. **(E)** CFHR4 expression was detected in WRL68, BEL7402, SK-Hep1, HCCLM3, HepG2, and Huh7 cell lines using Western blotting. **(F)** CFHR4 protein expression in 30 paired adjacent noncancerous tissues and HCC tissues. **(G)** CFHR4 expression was detected in WRL68, BEL7402, SK-Hep1, HCCLM3, HepG2, and Huh7 cell lines using PCR. **(H)** CFHR4 mRNA expression in 30 paired adjacent noncancerous tissues and HCC tissues. **(I)** ROC curves were created to investigate the value of CFHR4 in identifying HCC tissues. **(J, K)** Volcano plots of the DEGs and heatmap showing the top 10 DEGs. *p < 0.05, **p < 0.01, ***p < 0.001, NS, no significance.

By analyzing GTEx and TCGA datasets, we investigated the CFHR4 mRNA levels across cancer types using the Wilcoxon rank sum test, including adrenocortical carcinoma (ACC), bladder urothelial carcinoma (BLCA), breast invasive carcinoma (BRCA), cervical squamous cell carcinoma and endocervical adenocarcinoma (CESC), cholangiocarcinoma (CHOL), colon adenocarcinoma (COAD), esophageal carcinoma (ESCA), glioblastoma multiforme (GBM), head and neck squamous cell carcinoma (HNSC), kidney chromophobe (KICH), kidney renal clear cell carcinoma (KIRC), kidney renal papillary cell carcinoma (KIRP), acute myeloid leukemia (LAML), brain lower grade glioma (LGG), liver hepatocellular carcinoma (LIHC), lung adenocarcinoma (LUAD), lung squamous cell carcinoma (LUSC), mesothelioma (MESO), ovarian serous cystadenocarcinoma (OV), pancreatic adenocarcinoma (PAAD), pheochromocytoma and paraganglioma (PCPG), prostate adenocarcinoma (PRAD), rectum adenocarcinoma (READ), stomach adenocarcinoma (STAD), skin cutaneous melanoma (SKCM), testicular germ cell tumors (TGCT), thyroid carcinoma (THCA), uterine corpus endometrial carcinoma (UCEC) and uterine carcinosarcoma (UCS). We found that CFHR4 expression was significantly decreased in LIHC and CHOL compared with normal tissues (**Figure 1A**). We obtained similar results from the Timer and GEPIA databases (**Supplementary Figures 1A, B**). According to the expression of CFHR4 in 374 HCC tissues and 50 normal liver tissues, we confirmed that the CFHR4 expression level was also noticeably decreased in HCC tissues (P<0.001) (**Figure 1B**). Furthermore, CFHR4 was underexpressed in the GSE14520 HCC cohort (P<0.001) (**Figure 1C**). Similar results were obtained for adjacent HCC tissues among the 50 matched HCC tissues and adjacent HCC tissues (P<0.05) (**Figure 1D**). We extracted protein from human normal hepatic cells (WRL68) and HCC cells (BEL7402, SK-hep1, HCCLM3, HepG2 and Huh7) and confirmed the low expression of CFHR4 in HCC cells using Western blot (**Figure 1E**). Subsequently, 30 pairs of HCC samples were validated, and similar conclusions were reached (**Figure 1F**). CFHR4 mRNA expression levels were further validated using quantitative real-time PCR analyses (P<0.001) (**Figures 1G, H**). In addition, we constructed the receiver operating characteristic (ROC) curve. The area under the curve (AUC) for CFHR4 was 0.698, and it has a significant diagnostic value for HCC (**Figure 1I**).

### Identification of DEGs in HCC

According to the CFHR4 expression level, we divided the data from patients with HCC into high and low CFHR4 expression groups for comparison. The DESeq2 package was used to infer CFHR4-associated genes and analyze the DEGs between the high and low expression groups. An adjusted p value < 0.05 and absolute log2-fold change > 1.5 were considered statistically significant. A total of 721 significant DEGs were identified. 113 DEGs were associated with the high CFHR4 expression group, and 608 DEGs were associated with the low CFHR4 expression group (**Figure 1J** and **Supplementary Table 2**). The top 10 DEGs were identified, further analyzed using HTSeq-Counts and sorted by relative expression (**Figure 1K**).

### GO and KEGG Enrichment Analyses

**Figure 2:**
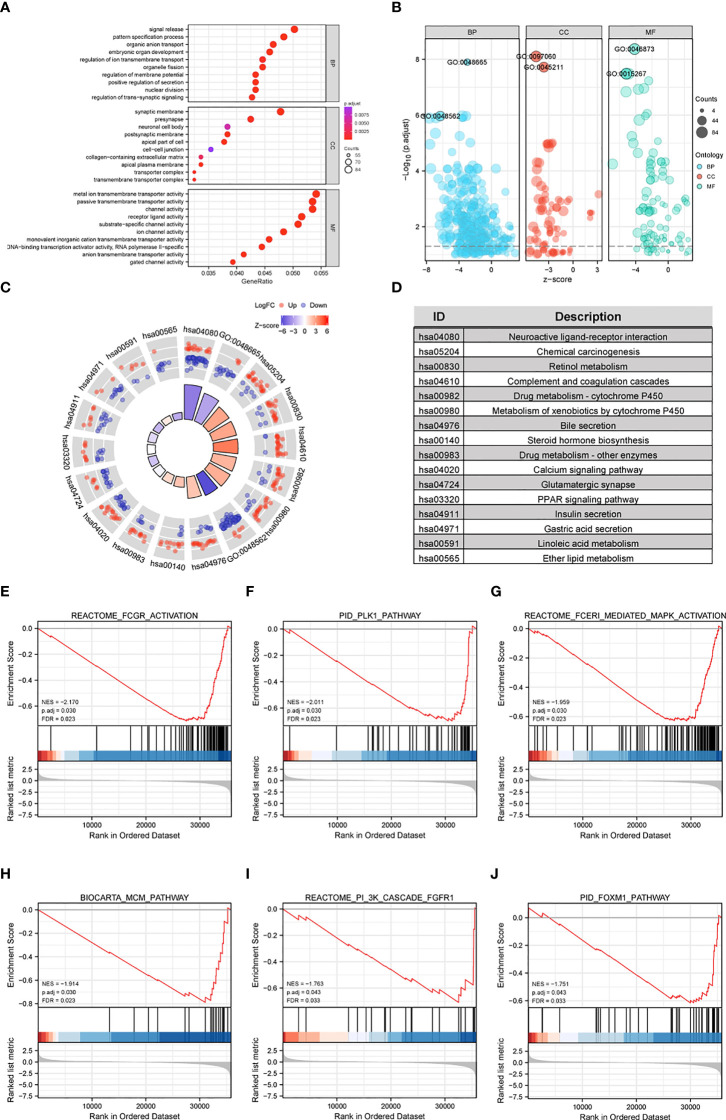
Functional enrichment analyses of CFHR4-related genes in HCC. **(A, B)** The enriched terms in GO categories in HCC. **(C, D)** KEGG pathway analysis based on CFHR4-associated DEGs. **(E–J)** GSEA enrichment plots, including FCGR, activated reaction, PLK1 pathway, reactant FCERI-mediated MAPK activation, ATR pathway, MCM pathway, cascade reactions of PI3K and FGFR1, reactant-mediated MAPK activation and FOXM1 pathway.

GO and KEGG enrichment analyses were performed using the “clusterProfiler” R package to further analyze the potential biological functions of CFHR4-related DEGs. The GO analysis indicated that CFHR4-related DEGs may be involved in gated channel activity, regulation of signal release, regulation of ion transmembrane transport, metal ion transmembrane transporter activity, synaptic membrane, transmembrane transporter complex and passive transmembrane transporter activity (**Figures 2A, B**; **Supplementary Table 3**). In the KEGG enrichment analysis, CFHR4-related DEGs were mainly involved in chemical carcinogenesis, retinol metabolism, the calcium signaling pathway, the PPAR signaling pathway, bile secretion, insulin secretion and gastric acid secretion (**Figures 2C, D**).

### CFHR4-Related Signaling Pathways Based on GSEA

GSEA was conducted between the high and low CFHR4 expression groups to further reveal CFHR4-related signaling pathways in HCC. The following pathways were significantly enriched in patients with low CFHR4 expression: FCGR-activated reaction, PLK1 pathway, reactant FCERI-mediated MAPK activation, ATR pathway, MCM pathway, cascade reaction of PI3K and FGFR1, reactant-mediated MAPK activation and FOXM1 pathway (**Figures 2E–J**; **Supplementary Table 4**).

### PPI Network Analysis

**Figure 3:**
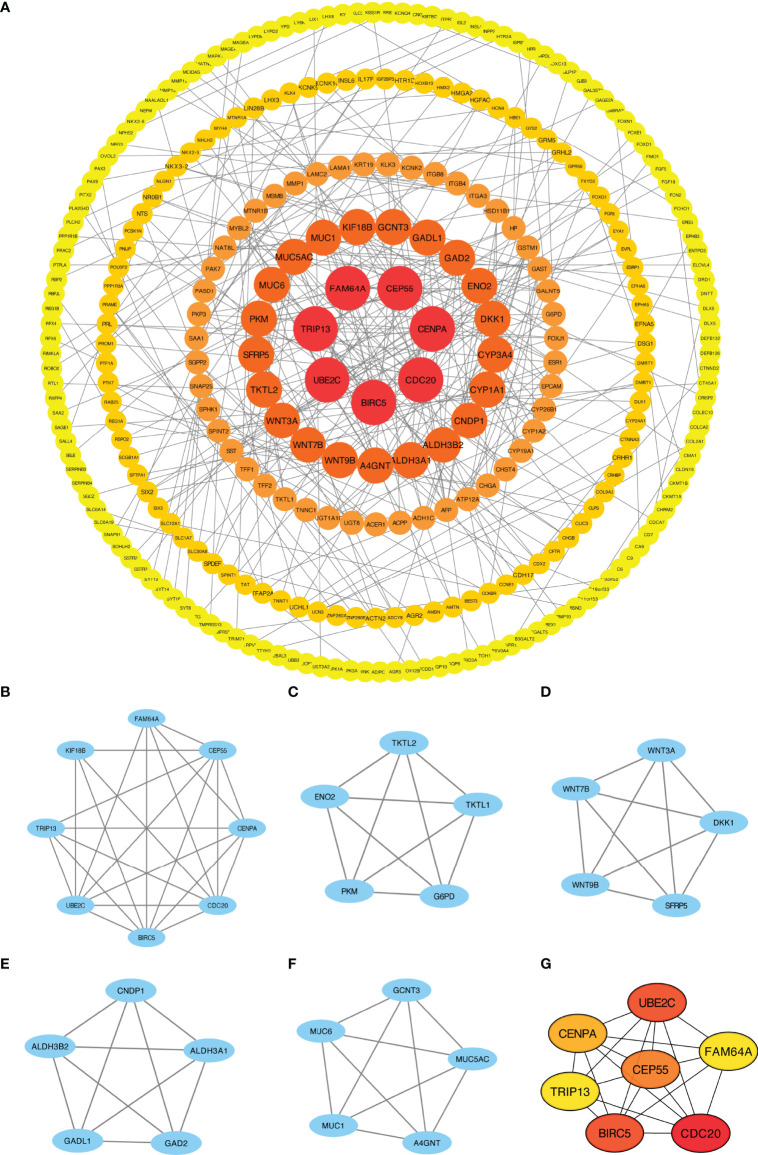
PPI network enrichment analysis. **(A)** The PPI network was built based on PPI pairs identified by the STRING dataset. **(B–F)** Hub gene clusters were selected from the PPI network (criteria of total scores ≥ 5,000). **(G)** Top 7 hub genes in the PPI network.

We explored the association between 721 DEGs in the HCC group using the STRING database by setting the interaction threshold to 0.70 and constructed a PPI network to further investigate the underlying mechanisms (**Figure 3A**; **Supplementary Table 5**). Subsequently, 301 proteins and 420 edges were screened, and five central gene clusters were identified using a total score ≥5000 (**Figures 3B–F**). In addition, the top 7 central genes were screened, including CENPA, CDC20, UBE2C, CEP55, BIRC5, FAM64A and TRIP13 (**Figure 3G**). By analyzing the GeneMANIA and STRING online datasets, potential CFHR4-interacting target genes were identified (**Supplementary Figures 2A, B**). CFHR4-related genes were selected by performing a crossover analysis, including C3, CRP, CFHR1, CFHR3 and CFHR5 (**Supplementary Figure 2C**). We subsequently analyzed the association between CFHR4 and the 5 intersecting genes (**Supplementary Figures 2D–H**).

### Correlation Between CFHR4 Expression and Immune Cell Infiltration

**Figure 4:**
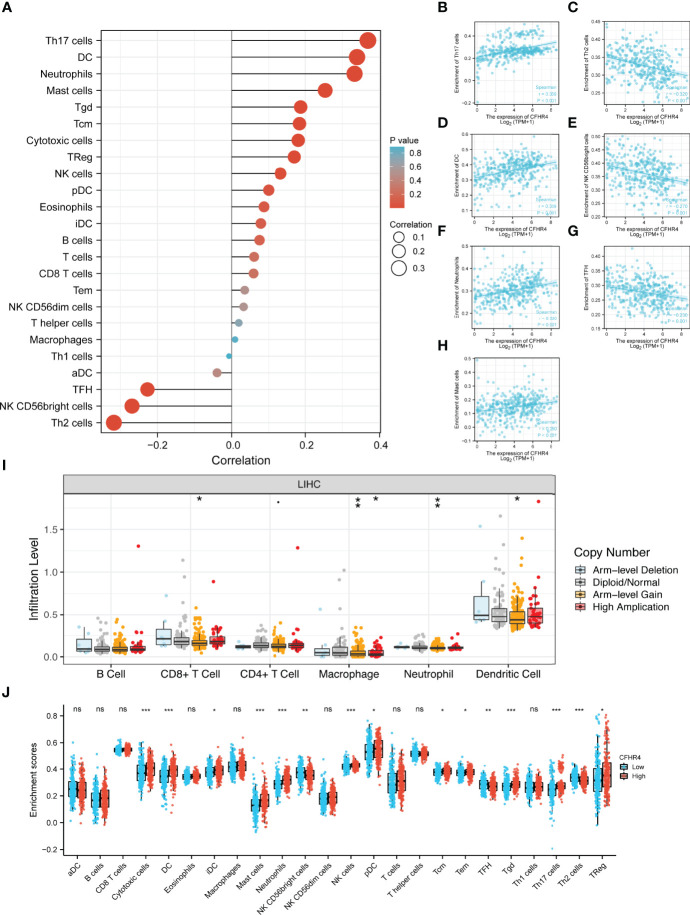
Integrative analysis of CFHR4 expression in the infiltrating immune microenvironment. **(A)** The forest plot depicts the relationship between the level of CFHR4 expression and the relative abundances of 24 immune cells. **(B–H)** Scatter plots showing the differentiation of Th17 cells, Th2 cells, DCs, NK CD56bright cells, neutrophils, TFH cells and mast cells infiltration levels between high and low groups of CFHR4 expression. **(I)** The SCNA showed that CFHR4 expression correlated with the level of immune cell infiltration. **(J)** Scatter plots showing the correlations between 24 immune cells and CFHR4 expression levels. *p < 0.05, **p < 0.01, ***p < 0.001, NS, no significance.

Based on the ssGSEA algorithm, we confirmed and quantified the correlations between CFHR4 expression and the immune cell infiltration levels (**Figure 4A**). The expression of CFHR4 was negatively correlated with aDCs, TFH cells, NK CD56bright cells and Th2 cells, and it has positive correlations with Th17 cells, DCs, neutrophils, mast cells, Tgd cells, Tcm cells, cytotoxic cells, Tregs, NK cells, pDCs, eosinophils, iDCs, B cells, T cells, CD8 T cells, Tems, NK CD56dim cells, T helper cells, macrophages and Th1 cells (**Figures 4B–H**). We further confirmed the correlation between CFHR4 expression with immunomarker of various immune cells in HCC. The results showed that CFHR4 expression was significantly correlated with the immunomarkers IRF5 and INOS of M1 macrophages in HCC (**Table 1**). It indicated that CFHR4 may induce macrophages to M1 polarization in HCC. This analysis of immune markers of different functions T cells showed that CFHR4 expression was highly correlated with the most immunomarkers (CD8B, CD3D, STAT1, IFN-γ, STAT5A, IL21, TGFβ, PD-1, CTLA4, LAG3 and TIM-3) of T cells in HCC (**Table 1**). It turns out that CFHR4 may perform an indispensable role in the T cells’ immune response to HCC. Especially for T cells exhaustion, consistent results with the GISTIC analysis were obtained. The somatic copy number alteration (SCNA) module demonstrated that the arm-level deletion of CFHR4 was markedly associated with immune cell infiltration levels in HCC (**Figure 4I**). In addition, the results also showed a correlation between CFHR4 expression and the immunomarkers of TAMs, neutrophils and dendritic cells (**Table 1**). Subsequently, according to the expression level of CFHR4, HCC samples were dichotomized into CFHR4-high and low expression groups, we aimed to reveal whether different expression groups of CFHR4 differ in the tumor immune microenvironment of HCC (**Figure 4J**). We found that cytotoxic cells, DCs, iDCs, mast cells, neutrophils, NK cells, pDCs, Tcm cells, Tem cells, Tgd cells, Th17 cells and Tregs were increased in the CFHR4 high expression group (P < 0.05), while the NK CD56bright cells, TFH cells and Th2 cells decreased (P < 0.05). These findings confirmed that reduced expression of CFHR4 in HCC was closely associated with immune cell infiltration.

**Table 1:** Correlation analysis between CFHR4 expression and biomarkers of immune cells.

Description	Gene markers	LIHC	
		Cor	P value
CD8+ T cell	CD8A	−0.074	0.152
	**CD8B**	−0.120	**0.017**
T cell (general)	**CD3D**	−0.200	**< 0.001**
	CD3E	−0.054	0.301
	CD2	−0.089	0.087
B cell	**CD19**	−0.140	**0.006**
	CD79A	−0.072	0.165
Monocyte	**CD86**	−0.170	**0.001**
	CD115 (CSF1R)	−0.072	0.165
TAM	CCL2	−0.005	0.922
	**CD68**	−0.210	**< 0.001**
	**IL10**	−0.110	**0.04**
M1 Macrophage	**INOS (NOS2)**	0.22	**< 0.001**
	**IRF5**	−0.230	**< 0.001**
	COX2 (PTGS2)	0.006	0.9
M2 Macrophage	CD163	0.079	0.129
	VSIG4	0.03	0.564
	MS4A4A	0.063	0.223
Neutrophils	**CD66b (CEACAM8)**	−0.120	**0.021**
	**CD11b (ITGAM)**	−0.130	**0.009**
	**CCR7**	0.12	**0.023**
Natural killer cell	KIR2DL1	0.064	0.215
	KIR2DL3	−0.047	0.367
	KIR2DL4	−0.069	0.183
	KIR3DL1	−0.009	0.866
	KIR3DL2	0.026	0.612
	KIR3DL3	−0.065	0.209
	KIR2DS4	0.005	0.929
	**HLA-DPB1**	−0.110	**0.038**
	HLA-DQB1	−0.043	0.411
	HLA-DRA	−0.003	0.956
	HLA-DPA1	0.051	0.327
	BDCA-1 (CD1C)	0.005	0.926
Dendritic cell	**BDCA-4 (NRP1)**	−0.110	**0.028**
	**CD11c (ITGAX)**	−0.160	**0.002**
Th1	T-bet (TBX21)	0.061	0.239
	STAT4	−0.091	0.078
	**STAT1**	−0.120	**0.016**
	**IFN-γ (IFNG)**	−0.110	**0.03**
	TNF-α (TNF)	−0.069	0.182
Th2	GATA3	−0.094	0.069
	STAT6	0.03	0.568
	**STAT5A**	−0.190	**< 0.001**
	IL13	−0.013	0.802
Tfh	BCL6	−0.022	0.669
	**IL21**	−0.110	**0.041**
	STAT3	0.082	0.113
	IL17A	0.035	0.496
Th17	FOXP3	0.08	0.123
	CCR8	−0.081	0.116
	STAT5B	−0.016	0.763
	**TGFβ (TGFB1)**	−0.260	**< 0.001**
T cell exhaustion	**PD-1 (PDCD1)**	−0.220	**< 0.001**
	**CTLA4**	−0.200	**< 0.001**
	**LAG3**	−0.240	**< 0.001**
	**TIM-3 (HAVCR2)**	−0.190	**< 0.001**
	GZMB	−0.086	0.098
Treg	FOXP3	0.08	0.123

The bold values indicates that the correlation analysis between CFHR4 and biomarker of immune cell is statistically significant.

### Correlation Between the CFHR4 Expression Level and Clinical Characteristics

**Figure 5:**
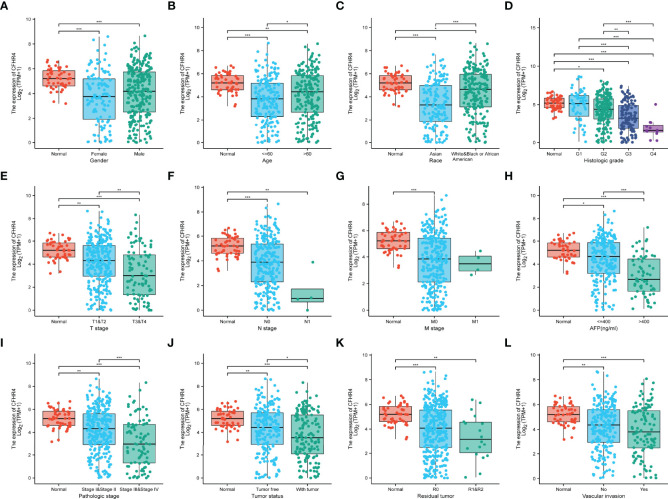
Correlation of CFHR4 expression with clinicopathological characteristics. **(A)** Sex. **(B)** Age. **(C)** Race. **(D)** Histologic grade. **(E)** T stage. **(F)** N stage. **(G)** M stage. **(H)** AFP level. **(I)** Pathological stage. **(J)** Tumor status. **(K)** Residual tumor. **(L)** Vascular invasion. *p < 0.05, **p < 0.01, ***p < 0.001.

The clinical data from patients with HCC in TCGA database were obtained to investigate the clinical characteristics of patients with different CFHR4 expression levels. After removing patients with incomplete clinical data, 374 patients remained for further analysis; the average age was 61.5 years (49.25 to 70.00 years), and 67% were male. **Table 2** provides a detailed description of the clinical data. We evaluated the differences in clinicopathological variables after stratifying patients based on CFHR4 expression using the Kruskal–Wallis test, and the level of CFHR4 was strongly correlated with age, sex, race, TNM stage, histologic grade, pathological stage, tumor status, residual tumor, vascular invasion and AFP level (**Figures 5A–L**). Notably, CFHR4 was expressed at higher levels in the older age group (age>60 years) than in the younger age group (age ≤ 60 years) (P<0.05). Significant differences in CFHR4 expression levels were also noted in different races (P<0.001). Moreover, a higher histological grade, TNM grade, pathological stage and tumor status were also significantly associated with low CFHR4 expression. Subsequently, we further confirmed the lower CFHR4 expression level in the group with a high AFP level (>400 ng/mL) (P<0.001). Based on these results, patients with HCC presenting lower CFHR4 expression seemed to have a more advanced tumor stage.

**Table 2:** The correlations between clinicopathological variables and CFHR4 expression.

Characteristic	Low expression of CFHR4	High expression of CFHR4	p
n	187	187	
Gender, n (%)			0.122
Female	68 (18.2%)	53 (14.2%)	
Male	119 (31.8%)	134 (35.8%)	
Race, n (%)			< 0.001
Asian	100 (27.6%)	60 (16.6%)	
Black or African American	6 (1.7%)	11 (3%)	
White	78 (21.5%)	107 (29.6%)	
Age, n (%)			0.011
<=60	101 (27.1%)	76 (20.4%)	
>60	85 (22.8%)	111 (29.8%)	
T stage, n (%)			0.017
T1	78 (21%)	105 (28.3%)	
T2	51 (13.7%)	44 (11.9%)	
T3	50 (13.5%)	30 (8.1%)	
T4	8 (2.2%)	5 (1.3%)	
N stage, n (%)			0.128
N0	136 (52.7%)	118 (45.7%)	
N1	4 (1.6%)	0 (0%)	
M stage, n (%)			0.628
M0	145 (53.3%)	123 (45.2%)	
M1	3 (1.1%)	1 (0.4%)	
Pathologic stage, n (%)			0.004
Stage I	74 (21.1%)	99 (28.3%)	
Stage II	45 (12.9%)	42 (12%)	
Stage III	55 (15.7%)	30 (8.6%)	
Stage IV	4 (1.1%)	1 (0.3%)	
Tumor status, n (%)			0.001
Tumor free	85 (23.9%)	117 (33%)	
With tumor	92 (25.9%)	61 (17.2%)	
Residual tumor, n (%)			0.321
R0	164 (47.5%)	163 (47.2%)	
R1	11 (3.2%)	6 (1.7%)	
R2	0 (0%)	1 (0.3%)	
Histologic grade, n (%)			< 0.001
G1	17 (4.6%)	38 (10.3%)	
G2	77 (20.9%)	101 (27.4%)	
G3	80 (21.7%)	44 (11.9%)	
G4	11 (3%)	1 (0.3%)	
AFP (ng/ml), n (%)			< 0.001
<=400	87 (31.1%)	128 (45.7%)	
>400	46 (16.4%)	19 (6.8%)	

### Prognostic Potential of CFHR4 in HCC

**Figure 6:**
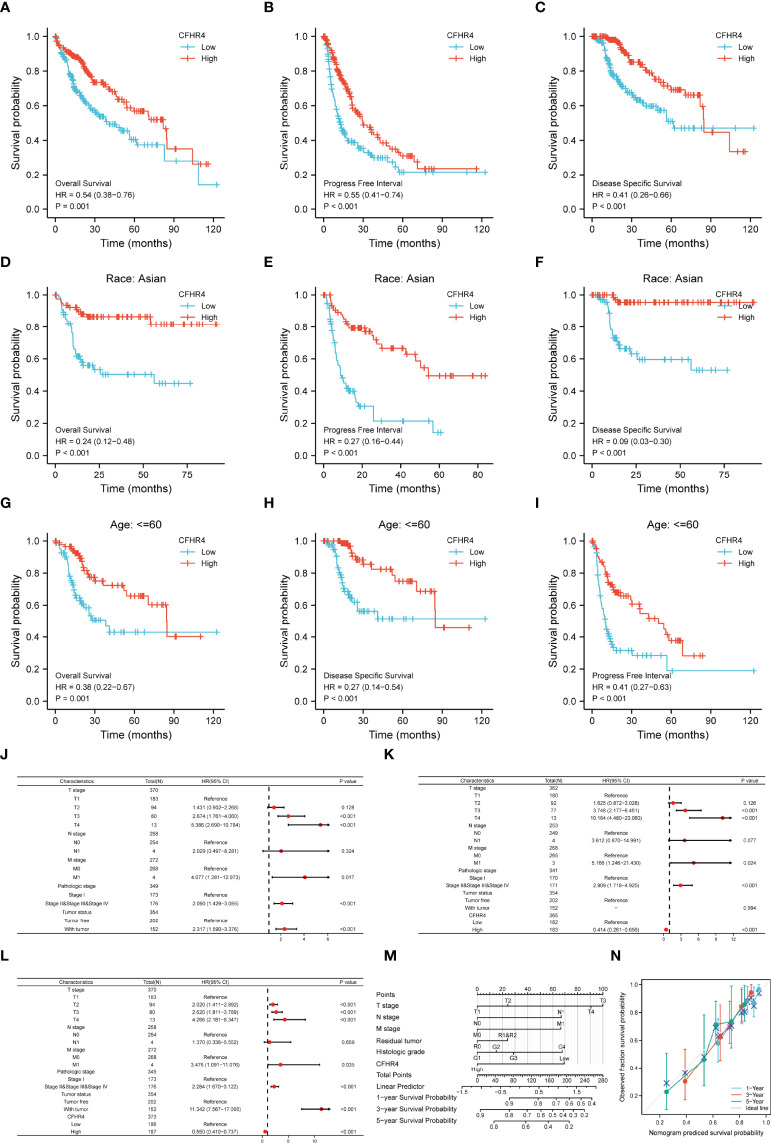
The prognostic value of CFHR4 in HCC. **(A–C)** Survival curves showing a comparison of OS, DSS and PFI between patients with HCC presenting high and low CFHR4 expression. **(D–F)** OS, DSS and PFI survival curves for Asian patients with HCC presenting high and low CFHR4 expression. **(G–I)** OS, DSS and PFI survival curves for patients with HCC aged ≤60 years presenting with high and low CFHR4 expression. **(J–L)** Univariate survival analysis of OS, PFI, and DSS in patients from different subgroups stratified according to TNM stage, pathological grade, tumor status, and CFHR4 expression levels. **(M)** For patients with HCC, a nomogram was constructed to estimate the probability of 1-, 3-, and 5-year OS. **(N)** Nomogram calibration plots for determining the probability of OS at 1, 3, and 5 years.

Afterward, we performed a series of studies to determine the association of CFHR4 expression levels with the prognosis of patients with HCC. The Kaplan–Meier Plotter analysis revealed an association between low CFHR4 expression and a poor prognosis (**Figures 6A–C**). Moreover, we performed subgroup analyses of OS, DSS and PFI. Patients with high CFHR4 expression had a correspondingly better prognosis for OS, DSS and PFI in the Asian group (**Figures 6D–F**). However, OS, DSS and PFI in the white and black or African–American subgroups were not significantly different (**Supplementary Figures 3A–C**). In addition, patients with HCC presenting high CFHR4 expression who were aged ≤ 60 years experienced longer OS and DSS but had a worse prognosis in terms of PFI (**Figure 6G–I**). However, no significant differences were observed in the younger age subgroups for OS, DSS and PFI (age ≤ 60 years) (**Supplementary Figures 3D–F**). We further confirmed that the T3 and T4 subgroups and the stage III and stage IV subgroups experienced poorer OS (**Supplementary Figures 3G, H**).

A univariate Cox regression analysis was performed with TNM stage, pathological grade, tumor status and CFHR4 expression levels to further identify factors associated with different prognoses (**Supplementary Table 6**). The forest plot illustrated that low expression of CFHR4 was a risk factor for the OS (**Figure 6J**; **Supplementary Table 7**), DSS (**Figure 6K**; **Supplementary Table 6**) and PFI (**Figure 6L**; **Supplementary Table 8**) of patients with HCC. According to the results of the univariate Cox regression analysis, CFHR4 expression and other independent clinicopathological factors were used to construct the point scale of the nomogram. Each variable was scored with reference to the scale of the nomogram, and the total scores were dispatched to the outcome line and predicted the prognosis of patients with at 1, 3 and 5 years. The C-index of the nomogram was 0.706 (95% confidence interval: 0.671-0.741). This result suggested that the prognostic nomogram of CFHR4 had good discriminatory power (**Figure 6M**). The deviation correction line in the calibration analysis approached the ideal curve, indicating that the predicted values were consistent with the observed values (**Figure 6N**). Consistent results were obtained with the univariate Cox regression analysis.

### CFHR4 Expression Is in Associated With m6A RNA Methylation Regulators in HCC

**Figure 7:**
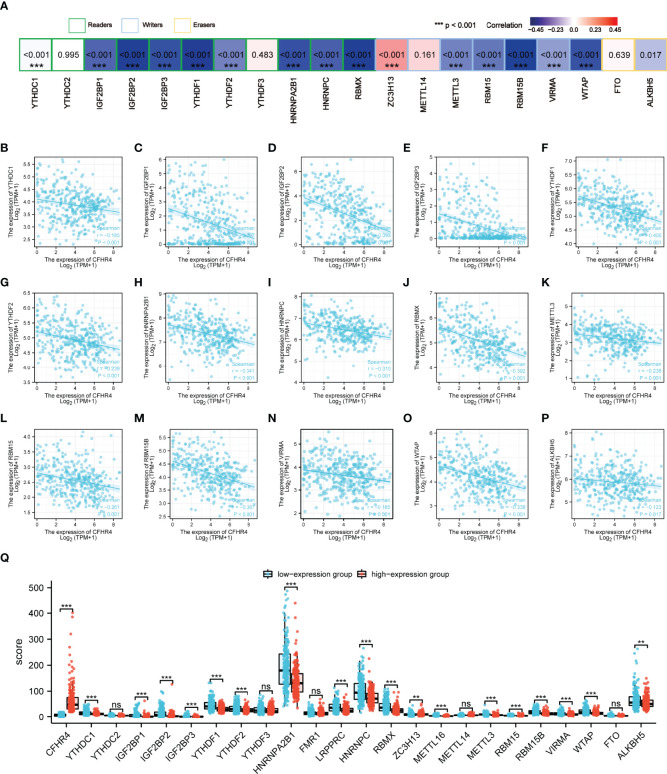
Analysis of the association between the CFHR4 expression level and the expression of m6A-related genes in HCC. **(A)** Correlation of CFHR4 expression levels with m6A gene expression in HCC. **(B–P)** Scatter plot showing the relationship between CFHR4 and m6A genes. **(Q)** Correlation of m6A genes in the CFHR4 high and low expression groups of HCC tumor samples. **p < 0.01, ***p < 0.001, NS, no significance.

As reported in previous studies, m6A RNA methylation exerts an important effect on the development of HCC (27–29). The correlations between CFHR4 expression and the expression of 23 m6A-related genes were analyzed in TCGA (**Figure 7A**). The correlation analysis showed significant negative correlations between the expression of CFHR4 (P < 0.05) and 15 m6A-related genes in HCC (**Figures 7B–P**). Furthermore, groups were established based on the median CFHR4 expression, and 211 patients were assigned to the high expression group and 210 patients were assigned to in the low expression group. We determined the relationship between the CFHR4 expression level and m6A modification level in HCC by analyzing the differential expression of 23 m6A-related genes in different expression groups (**Figure 7Q**). The expression of YTHDC1, IGF2BP1, IGF2BP2, IGF2BP3, YTHDF1, YTHDF2, HNRNPA2B1, LRPPRC, HNRNPC, RBMX, METTL16, METTL3, RBM15, RBM15B, VIRMA, WTAP and ALKBH5 was reduced in the high CFHR4 expression group (P < 0.05). In summary, a strong correlation was observed between m6A RNA methylation in HCC and the CFHR4 expression level.

### Construction of a CFHR4-Related ceRNA Triple Regulatory Network

**Figure 8:**
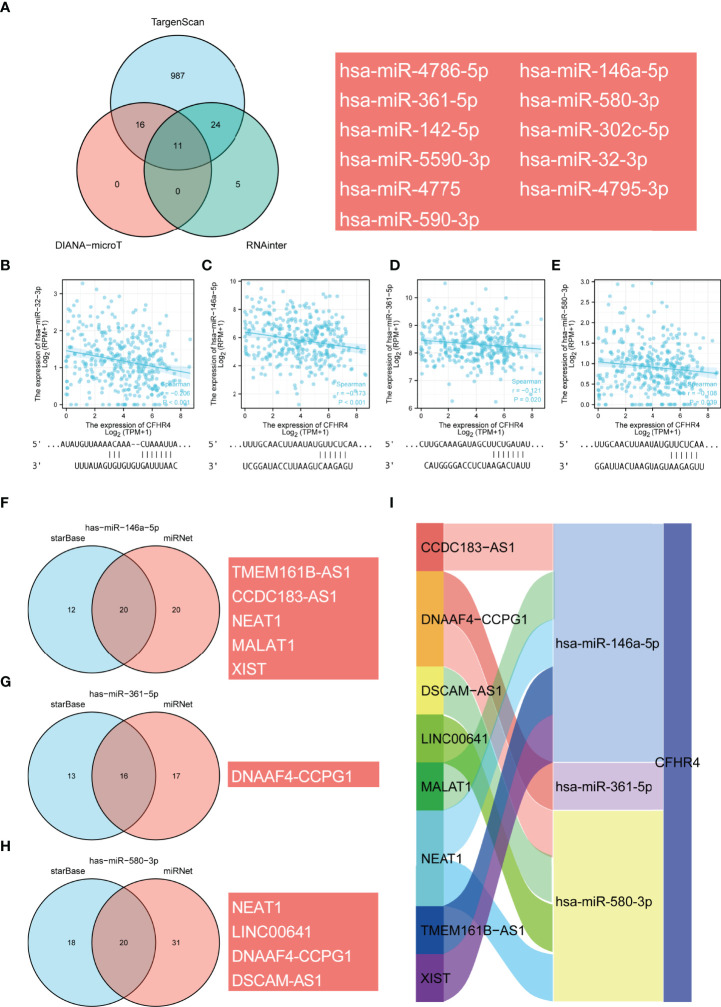
Prediction of the ceRNA network in HCC. **(A)** Venn diagram showing the results for CFHR4 targets predicted using the TargetScan, DIANA-microT and RNAinter databases. **(B–E)** Scatter plots were generated to show miRNAs-mRNAs with significant correlations. TargetScan prediction of the potential binding sites in CFHR4 for the target miRNAs. **(F–H)** The lncRNAs that bind to target miRNAs were predicted using the miRNet and starBase online databases and displayed in a Venn diagram, including hsa−miR−146−5p, hsa−miR−361−5p and hsa−miR−580−3p. **(I)** Sankey diagram showing the CFHR4-related ceRNA regulatory network.

Accumulating evidence highlights the regulatory role of lncRNA–miRNA–mRNA ceRNA networks in cancers. Therefore, we analyzed and constructed a ceRNA regulatory network for CFHR4 in HCC. Through TargetScan, DIANA-microT and RNAinter database predictions, the following 11 miRNAs were jointly predicted: hsa-miR-32-3p, hsa-miR-142-5p, hsa-miR-146a-5p, hsa-miR-302c-5p, hsa-miR-361-5p, hsa-miR-4775, hsa-miR-4786-5p, hsa-miR-4795-3p, hsa-miR-5590-3p, hsa-miR-580-3p and hsa-miR-590-3p (**Figure 8A**). Based on the regulatory relationship in the ceRNA network, a negative correlation was observed between mRNAs and miRNAs. Four miRNAs negatively correlated with CFHR4 expression were identified and screened by performing a correlation analysis. The scatter plots showed the correlation between CFHR4 expression and the target miRNAs, and the TargetScan database was used to predict the potential binding sites in CFHR4 for target miRNAs (**Figures 8B–E**). Subsequently, the lncRNAs that may interact with the target miRNAs (hsa-miR-146a-5p, hsa-miR-361-5p and hsa-miR-580-3p) were further predicted using the miRNet and starBase databases (**Figures 8F–H**). This interaction is due to the negative correlation between the expression of lncRNAs and miRNAs. Consequently, using the starBase database, we further screened and confirmed the lncRNAs in HCC that were negatively correlated with the three target miRNAs. Based on these results, the following 10 ceRNA regulatory networks that play a role in HCC were constructed: TMEM161B-AS1-hsa-miR-146a-5p-CFHR4, CCDC183-AS1-hsa-miR-146a-5p-CFHR4, NEAT1-hsa-miR-146a-5p-CFHR4, MALAT1-hsa-miR-146a-5p-CFHR4, XIST-hsa-miR-146a-5p-CFHR4, DNAAF4-CCPG1-hsa-miR-361-5p-CFHR4, NEAT1-hsa-miR-580-3p-CFHR4, LINC00641-hsa-miR-580-3p-CFHR4, DNAAF4-CCPG1-hsa-miR-580-3p-CFHR4 and DSCAM-AS1-hsa-miR-580-3p-CFHR4 (**Figure 8I**).

## Discussion

The CFHR family consists of five highly related proteins. Each CFHR gene has a completely duplicated structural domain in the plasma proteins, and they share high sequence identity (8, 9). Members of the CFHR family of proteins play key roles in the progression of multiple diseases through multiple mechanisms. For example, CFHR1 exacerbates atherosclerotic cardiovascular disease by altering the expression levels of C-reactive protein apolipoprotein and serum amyloid protein A (30). All CFHR genes are genetic risk factors for AMD (31). The CFHR family of genes is also important in AHUS and C3 glomerulopathy (11, 13, 32). In addition, some members of the CFHR family of proteins have been proven to exert a marked effect on the progression of a variety of cancers (33–35). However, few studies on CFHR4 have been conducted, and no studies have determined its role in cancer.

In the present study, we measured the expression level and prognostic value of CFHR4. We confirmed that CFHR4 mRNA expression was markedly downregulated in HCC and CHOL tissues, and these results were validated in multiple databases. The ROC curve analysis suggested that CFHR4 may be a promising diagnostic biomarker for differentiating HCC from normal tissue.

We confirmed the reduced expression of CFHR4 in HCC cell lines and HCC samples by performing *in vitro* experiments. We analyzed the DEGs related to CFHR4 to further assess the role of CFHR4 in HCC. By conducting GO and KEGG analyses, we found that differences in CFHR4 expression were significantly correlated with regulating signal release, regulation of ion transmembrane transport, gated channel activity, metal ion transmembrane transporter activity, calcium signaling pathway and the PPAR signaling pathway. Using GSEA, we also revealed that low CFHR4 expression was significantly associated with FCGR-activated reactions, the PLK1 pathway, reactant FCERI-mediated MAPK activation, the ATR pathway, the MCM pathway, the cascade reactions of PI3K and FGFR1, reactant-mediated MAPK activation and the FOXM1 pathway in patients. PLK1 (36), MAPK (37), ATR (38), MCM (39), PI3K and FGFR1 (40) have been shown to play increasingly crucial regulatory roles in HCC, and these studies and our results indicated that CFHR4 may inhibit the development and progression of HCC by regulating these signaling pathways. However, the association of CFHR4 with these signaling pathways was first discovered here, and the regulatory mechanisms require further exploration. Furthermore, based on the DEGs, we constructed the PPI networks using the Cytoscape tool. Five central gene clusters (a total score ≥ 5000) and the top 7 central genes were screened, including CENPA, CDC20, UBE2C, CEP55, BIRC5, FAM64A and TRIP13. The CFHR4-interacting genes were generated using STRING and GeneMANIA online databases, and we observed five intersecting genes, including C3, CRP, CFHR1, CFHR3 and CFHR5. Existing studies have confirmed that CFHR4 regulates complement activation and opsonization on biological surfaces by interacting with native CRP (Hebecker et al., 2010). CFHR4 interacts with C3b (C3 activation fragment) (Hellwage et al., 1999, Hebecker and Jozsi, 2012). These conclusions promote the credibility of the predictions from the STRING database and will provide critical insights into the design of follow-up studies and experimental validation.

Among the results, tumor infiltrating immune cells (TIICs) were recently shown to play a pivotal regulatory role in tumor progression (41). The substantial accumulation of TIICs in HCC affects the prognosis of HCC (42). By revealing the relationship between CFHR4 expression and the level of immune cell infiltration in HCC, CFHR4 expression was clearly associated with the infiltration of Th17 cells, DCs, neutrophils and Th2 cells. Th17 cells are a major effector subset of CD4+ T cells that play a vital role in host protection and autoinflammatory disorders (43, 44). The differentiation of Th17 cells into Th1 and Th2 cell subsets participates in regulating the response to intracellular pathogens and extracellular organisms (45). Th1/17 cells produce IFN-γ to drive antitumor immune responses (46). Multiple studies reported that increased infiltration of Th17 cells inhibits the progression of breast cancer (47). Moreover, DCs are specialized antigen-presenting cells that play important roles in the initiation and regulation of innate and adaptive immune responses (48). The antitumor effect of DCs has been confirmed (49). Neutrophils have also been proven to exert bidirectional regulatory effects on the tumor immune microenvironment (50). Our studies indicated that high CFHR4 expression activated Th17 cells, DCs and neutrophils to promote antitumor immune responses. In addition, antigen-presenting cells might promote the polarization of CD4+ T cells toward Th1 and Th2 cell subsets. Th1 cells are mainly involved in cellular immunity and tumor clearance, and Th2 cells are involved in the stimulation of antibody production (51). Th2 cells have also been confirmed as an independent risk factor for cancer growth and progression (52, 53). The number of NK CD56bright cells is significantly increased in various cancers (54–56). Multiple studies reported that Tfh cells are a specialized subset of CD4+ T cells that support the germinal centers, which secrete high-affinity antibodies and provide help for memory B cells (57, 58). Additionally, Tfh cells were confirmed to be involved in human autoimmune responses and cancers (59, 60). Based on this information, CFHR4 modulates immune responses mediated by Th2 cells, NK CD56bright cells and Tfh cells in HCC. We also found that the CFHR4 CNV was significantly correlated with the levels of infiltrating CD8+ T cells, macrophages, neutrophils, and dendritic cells. In addition, CFHR4 expression is strongly correlated with various immunomarker groups in HCC. We confirmed significant correlations between CFHR4 expression and CD8+ T cells (CD8B), monocytes (CD86), TAMs (CD68 and IL10), M1 macrophages (NOS2 and IRF5), neutrophils (CD66b, CD11b, and CCR7), natural killer cells (HLA-DPB1), dendritic cells (NRP1 and ITGAX), Th1 cells (STAT1 and IFN-γ), Th2 cells (STAT5A), Tfh cells (IL21), Th17 cells (TGFβ) and exhausted T cells (PD-1, CTLA4, LAG3, and TIM-3). Our identified a potentially indispensable role for CFHR4 in regulating immune cell infiltration in HCC. We explored the relationship between CFHR4 expression with OS, PFI, DSS and clinical characteristics (TNM stage, residual tumor, and histological grade) by performing univariate Cox regression analysis. Calibration plots showed good agreement between predicted values of CFHR4-related column line plots and forecasted and observed values for 1-, 3- and 5-year OS probabilities. These results were consistent with those of the univariate Cox regression analysis.

The m6A methylation exerts a substantial effect on tumor cell proliferation, invasion and migration (61). Currently, m6A RNA and ceRNA regulatory networks are widely studied to determine HCC mechanisms (15). We further analyzed the relationship between CFHR4 expression and m6A modifications and determined that CFHR4 expression had inseparable relationships with IGF2BP2, IGF2BP3, YTHDF1, HNRNPA2B1, LRPPRC, HNRNPC, RBMX, RBM15B and WTAP expression. We also observed significant correlations between high CFHR4 expression and YTHDC1, IGF2BP1, IGF2BP2, IGF2BP3, YTHDF1, YTHDF2, HNRNPA2B1, LRPPRC, HNRNPC, RBMX, METTL16, METTL3, RBM15, RBM15B, VIRMA, WTAP and ALKBH5 expression. Multiple studies have now reported that IGF2BP1 (62), IGF2BP2 (28), IGF2BP3 (63), YTHDF1 (64), YTHDF2 (29), BRMX (65), RBM15 (66), METTL3 (67) and WTAP (27) are significantly upregulated in HCC, and their overexpression promotes HCC progression and is associated with a poor prognosis for patients with HCC. These discussions further supported our results. Thus, these findings suggested that the CFHR4 gene may be modified by m6A to increase the stability of its mRNA, which further inhibits the proliferation, invasion and migration of HCC. Subsequently, we constructed ceRNA regulatory networks based on the prediction. Because the ceRNA regulatory networks of CFHR4 were derived from a bioinformatics analysis, more experiments are needed to validate this network in future studies.

Although we increased our awareness of the regulatory mechanism of CFHR4 in HCC, the study had several limitations. Initially, the expression levels of CFHR4 and the important regulatory mechanisms and pathways related to CFHR4 in HCC should be further validated and evaluated by analyzing clinical samples from more centers. Secondly, However, the potential diagnostic value of the circulating CFHR4 content in HCC patients is not clear, and the clinical significance of circulating tumor markers remains to be further explored. In addition, the relationship between CFHR4 and interacting genes and m6A genes in HCC should be further explored and validated. In future studies, we will further elucidate the potential regulatory mechanisms of CFHR4 in HCC by performing more experiments.

## Conclusions

In summary, this study represents the first in-depth analysis of CFHR4 in HCC. Our study suggested that CFHR4 was abnormally downregulated in HCC and that its reduced expression was correlated with a poorer prognosis. We confirmed the correlation between CFHR4 expression and the m6A modification, indicating that CFHR4 may be modified by m6A to improve mRNA stability. The construction of ceRNA networks suggested that CFHR4 may be involved in multiple molecular regulatory mechanisms of HCC. More importantly, CFHR4 expression was associated with multiple immune cells and may affect HCC tumor immunity by inducing M1 macrophage polarization and altering the infiltration of exhausted T cells. These findings provide additional insights into the mechanism by which CFHR4 may represent an important independent prognostic marker for HCC. The potential molecular mechanisms and regulatory networks of CFRH4 provide a basis for follow-up studies. The study also provides important insights into the treatment of HCC based on genomics.

## Data Availability Statement

The article/**Supplementary Material** contains the original contributions presented in the study. Any additional questions can be forwarded to the corresponding authors.

## Ethics Statement

The Ethics Committee of the First Affiliated Hospital of Harbin Medical University provided ethical review and approval. The patients provided their written informed consent to participate in this study.

## Author Contributions

HY, CW, SK, and MB made equal contributions to this article. HY, CW, and SK planned the research trials and engaged in article writing. YNX, SL, ZF, BQ, YX, and YF attended in information generation and analysis. MZ, ZL, BY, XL, YH, YZ, and SP also provided assistance with analysis. All authors made great efforts to the article and agreed to the version submitted.

## Funding

This work was supported by the Research Fund of the National Natural Scientific Foundation of China (81100305, 81470876 and 81270527), Natural Science Foundation of Heilongjiang Province of China (QC2013C094, LC2018037), Chen Xiaoping Foundation for the Development of Science and Technology of Hubei Province (CXPJJH11900001-2019349), Outstanding Youth Training Fund from Academician Yu Weihan of Harbin Medical University (2014), and the First Affiliated Hospital of Harbin Medical University (2019L01, HYD2020JQ0007).

## Conflict of Interest

The authors declare that the research was conducted in the absence of any commercial or financial relationships that could be construed as a potential conflict of interest.

## Publisher’s Note

All claims expressed in this article are solely those of the authors and do not necessarily represent those of their affiliated organizations, or those of the publisher, the editors and the reviewers. Any product that may be evaluated in this article, or claim that may be made by its manufacturer, is not guaranteed or endorsed by the publisher.

## Supplementary Material

Supplementary Figure 1 CFHR4 expression levels in different cancer tissues compared to normal tissues. **(A)** CFHR4 expression levels in different cancer tissues compared to normal tissues in the Timer database. **(B)** CFHR4 expression levels in different cancer tissues compared to normal tissues in the GEPIA database. *p < 0.05, **p < 0.01, ***p < 0.001, NS, no significance.

Supplementary Figure 2 PPI network and potential CFHR4-interacting target genes. **(A)** PPI networks were built using the STRING database. **(B)** PPI networks were built using the GeneMANIA database. **(C)** The intersecting genes identified by the STRING and GeneMANIA online databases are displayed in a Venn diagram. **(D)** Scatter plot showing the correlation between CFHR1 and CFHR4 expression. **(E)** Scatter plot showing the correlation between CFHR3 and CFHR4 expression. **(F)** Scatter plot showing the correlation between CFHR5 and CFHR4 expression. **(G)** Scatter plot showing the correlation between C3 and CFHR4 expression. **(H)** Scatter plot showing the correlation between CRP and CFHR4 expression.

Supplementary Figure 3 The prognostic value of CFHR4 in HCC. **(A–C)** OS, DSS and PFI survival curves for white, black or African–American patients with HCC presenting high and low CFHR4 expression. **(D-F)** OS, DSS and PFI survival curves for patients with HCC aged > 60 years presenting with high and low CFHR4 expression. **(G)** OS curves for patients with stage T3 and T4 HCC presenting with high and low CFHR4 expression. **(H)** OS curves for patients with pathological stage III and IV HCC presenting with high and low CFHR4 expression.

Supplementary Table 1 Characteristics of patients with HCC in TCGA.

Supplementary Table 2 CFHR4-related DEGs.

Supplementary Table 3 GO enrichment analysis of CFHR4.

Supplementary Table 4 GSEA enrichment analysis of CFHR4.

Supplementary Table 5 PPI network of CFHR4.

Supplementary Table 6 OS of patients with HCC based on prognostic covariates.

Supplementary Table 7 DSS patients with HCC based on prognostic covariates.

Supplementary Table 8 PFI of patients with HCC based on prognostic covariates.

## References

[1] Sung H Ferlay J Siegel RL Laversanne M Soerjomataram I Jemal A. Global Cancer Statistics 2020: GLOBOCAN Estimates of Incidence and Mortality Worldwide for 36 Cancers in 185 Countries. CA Cancer J Clin (2021) 71(3):209–49. doi: 10.3322/caac.21660 33538338

[2] Chen W Zheng R Baade PD Zhang S Zeng H Bray F. Cancer Statistics in China, 2015. CA Cancer J Clin (2016) 66(2):115–32. doi: 10.3322/caac.21338 26808342

[3] Forner A Reig M Bruix J. Hepatocellular Carcinoma. Lancet (2018) 391(10127):1301–14. doi: 10.1016/S0140-6736(18)30010-2 29307467

[4] Singal AG Lampertico P Nahon P. Epidemiology and Surveillance for Hepatocellular Carcinoma: New Trends. J Hepatol (2020) 72(2):250–61. doi: 10.1016/j.jhep.2019.08.025 PMC6986771 31954490

[5] Wang C Dong L Li X Li Y Zhang B Wu H. The PGC1alpha/NRF1-MPC1 Axis Suppresses Tumor Progression and Enhances the Sensitivity to Sorafenib/Doxorubicin Treatment in Hepatocellular Carcinoma. Free Radic Biol Med (2021) 163:141–52. doi: 10.1016/j.freeradbiomed.2020.11.035 33276082

[6] Reis ES Mastellos DC Ricklin D Mantovani A Lambris JD. Complement in Cancer: Untangling an Intricate Relationship. Nat Rev Immunol (2018) 18(1):5–18. doi: 10.1038/nri.2017.97 28920587 PMC5816344

[7] Yarmoska SK Alawieh AM Tomlinson S Hoang KB. Modulation of the Complement System by Neoplastic Disease of the Central Nervous System. Front Immunol (2021) 12:689435. doi: 10.3389/fimmu.2021.689435 34671342 PMC8521155

[8] Poppelaars F Goicoechea de Jorge E Jongerius I Baeumner AJ Steiner MS Jozsi M. A Family Affair: Addressing the Challenges of Factor H and the Related Proteins. Front Immunol (2021) 12:660194. doi: 10.3389/fimmu.2021.660194 33868311 PMC8044877

[9] Skerka C Chen Q Fremeaux-Bacchi V Roumenina LT. Complement Factor H Related Proteins (CFHRs). Mol Immunol (2013) 56(3):170–80. doi: 10.1016/j.molimm.2013.06.001 23830046

[10] Lores-Motta L Paun CC Corominas J Pauper M Geerlings MJ Altay L. Genome-Wide Association Study Reveals Variants in CFH and CFHR4 Associated With Systemic Complement Activation: Implications in Age-Related Macular Degeneration. Ophthalmology (2018) 125(7):1064–74. doi: 10.1016/j.ophtha.2017.12.023 29398083

[11] Cipriani V Lores-Motta L He F Fathalla D Tilakaratna V McHarg S. Increased Circulating Levels of Factor H-Related Protein 4 Are Strongly Associated With Age-Related Macular Degeneration. Nat Commun (2020) 11(1):778. doi: 10.1038/s41467-020-14499-3 32034129 PMC7005798

[12] Zhao J Wu H Khosravi M Cui H Qian X Kelly JA. Association of Genetic Variants in Complement Factor H and Factor H-Related Genes With Systemic Lupus Erythematosus Susceptibility. PLoS Genet (2011) 7(5):e1002079. doi: 10.1371/journal.pgen.1002079 21637784 PMC3102741

[13] Zipfel PF Wiech T Stea ED Skerka C. CFHR Gene Variations Provide Insights in the Pathogenesis of the Kidney Diseases Atypical Hemolytic Uremic Syndrome and C3 Glomerulopathy. J Am Soc Nephrol (2020) 31(2):241–56. doi: 10.1681/ASN.2019050515 PMC7003313 31980588

[14] Moore I Strain L Pappworth I Kavanagh D Barlow PN Herbert AP. Association of Factor H Autoantibodies With Deletions of CFHR1, CFHR3, CFHR4, and With Mutations in CFH, CFI, CD46, and C3 in Patients With Atypical Hemolytic Uremic Syndrome. Blood (2010) 115(2):379–87. doi: 10.1182/blood-2009-05-221549 PMC2829859 19861685

[15] Wang P Wang X Zheng L Zhuang C. Gene Signatures and Prognostic Values of M6a Regulators in Hepatocellular Carcinoma. Front Genet (2020) 11:540186. doi: 10.3389/fgene.2020.540186 33133142 PMC7567013

[16] Yang Y Hsu PJ Chen YS Yang YG. Dynamic Transcriptomic M(6)A Decoration: Writers, Erasers, Readers and Functions in RNA Metabolism. Cell Res (2018) 28(6):616–24. doi: 10.1038/s41422-018-0040-8 PMC5993786 29789545

[17] Zaccara S Ries RJ Jaffrey SR. Reading, Writing and Erasing mRNA Methylation. Nat Rev Mol Cell Biol (2019) 20(10):608–24. doi: 10.1038/s41580-019-0168-5 31520073

[18] Pan Y Xiao K Li Y Li Y Liu Q. RNA N6-Methyladenosine Regulator-Mediated Methylation Modifications Pattern and Immune Infiltration Features in Glioblastoma. Front Oncol (2021) 11:632934. doi: 10.3389/fonc.2021.632934 33718217 PMC7947873

[19] Zhang F Luo BH Wu QH Li QL Yang KD. LncRNA HCG18 Upregulates TRAF4/TRAF5 to Facilitate Proliferation, Migration and EMT of Epithelial Ovarian Cancer by Targeting miR-29a/B. Mol Med (2022) 28(1):2. doi: 10.1186/s10020-021-00415-y 34983361 PMC8725507

[20] Xue ST Zheng B Cao SQ Ding JC Hu GS Liu W. Long non-Coding RNA LINC00680 Functions as a ceRNA to Promote Esophageal Squamous Cell Carcinoma Progression Through the miR-423-5p/PAK6 Axis. Mol Cancer (2022) 21(1):69. doi: 10.1186/s12943-022-01539-3 35255921 PMC8900330

[21] Li D Xu M Wang Z Huang P Huang C Chen Z. The EMT-Induced lncRNA NR2F1-AS1 Positively Modulates NR2F1 Expression and Drives Gastric Cancer *via* miR-29a-3p/VAMP7 Axis. Cell Death Dis (2022) 13(1):84. doi: 10.1038/s41419-022-04540-2 35082283 PMC8791943

[22] Love MI Huber W Anders S. Moderated Estimation of Fold Change and Dispersion for RNA-Seq Data With Deseq2. Genome Biol (2014) 15(12):550. doi: 10.1186/s13059-014-0550-8 25516281 PMC4302049

[23] Subramanian A Tamayo P Mootha VK Mukherjee S Ebert BL Gillette MA. Gene Set Enrichment Analysis: A Knowledge-Based Approach for Interpreting Genome-Wide Expression Profiles. Proc Natl Acad Sci USA (2005) 102(43):15545–50. doi: 10.1073/pnas.0506580102 PMC1239896 16199517

[24] Yu G Wang LG Han Y He QY. Clusterprofiler: An R Package for Comparing Biological Themes Among Gene Clusters. OMICS (2012) 16(5):284–7. doi: 10.1089/omi.2011.0118 PMC3339379 22455463

[25] Hanzelmann S Castelo R Guinney J. GSVA: Gene Set Variation Analysis for Microarray and RNA-Seq Data. BMC Bioinf (2013) 14:7. doi: 10.1186/1471-2105-14-7 PMC3618321 23323831

[26] Bindea G Mlecnik B Tosolini M Kirilovsky A Waldner M Obenauf AC. Spatiotemporal Dynamics of Intratumoral Immune Cells Reveal the Immune Landscape in Human Cancer. Immunity (2013) 39(4):782–95. doi: 10.1016/j.immuni.2013.10.003 24138885

[27] Chen Y Peng C Chen J Chen D Yang B He B. WTAP Facilitates Progression of Hepatocellular Carcinoma *via* M6a-HuR-Dependent Epigenetic Silencing of ETS1. Mol Cancer (2019) 18(1):127. doi: 10.1186/s12943-019-1053-8 31438961 PMC6704583

[28] Pu J Wang J Qin Z Wang A Zhang Y Wu X. IGF2BP2 Promotes Liver Cancer Growth Through an M6a-FEN1-Dependent Mechanism. Front Oncol (2020) 10:578816. doi: 10.3389/fonc.2020.578816 33224879 PMC7667992

[29] Zhang C Huang S Zhuang H Ruan S Zhou Z Huang K. YTHDF2 Promotes the Liver Cancer Stem Cell Phenotype and Cancer Metastasis by Regulating OCT4 Expression *via* M6a RNA Methylation. Oncogene (2020) 39(23):4507–18. doi: 10.1038/s41388-020-1303-7 32366907

[30] Irmscher S Zipfel SLH Halder LD Ivanov L Gonzalez-Delgado A Waldeyer C. Factor H-Related Protein 1 (FHR-1) Is Associated With Atherosclerotic Cardiovascular Disease. Sci Rep (2021) 11(1):22511. doi: 10.1038/s41598-021-02011-w 34795372 PMC8602345

[31] Cipriani V Tierney A Griffiths JR Zuber V Sergouniotis PI Yates JRW. Beyond Factor H: The Impact of Genetic-Risk Variants for Age-Related Macular Degeneration on Circulating Factor-H-Like 1 and Factor-H-Related Protein Concentrations. Am J Hum Genet (2021) 108(8):1385–400. doi: 10.1016/j.ajhg.2021.05.015 PMC8387294 34260948

[32] Zuber J Frimat M Caillard S Kamar N Gatault P Petitprez F. Use of Highly Individualized Complement Blockade Has Revolutionized Clinical Outcomes After Kidney Transplantation and Renal Epidemiology of Atypical Hemolytic Uremic Syndrome. J Am Soc Nephrol (2019) 30(12):2449–63. doi: 10.1681/ASN.2019040331 PMC6900783 31575699

[33] Riihila PM Nissinen LM Ala-Aho R Kallajoki M Grenman R Meri S. Complement Factor H: A Biomarker for Progression of Cutaneous Squamous Cell Carcinoma. J Invest Dermatol (2014) 134(2):498–506. doi: 10.1038/jid.2013.346 23938460

[34] Fan WL Yang LY Hsieh JC Lin TC Lu MJ Liao CT. Prognostic Genetic Biomarkers Based on Oncogenic Signaling Pathways for Outcome Prediction in Patients With Oral Cavity Squamous Cell Carcinoma. Cancers (Basel) (2021) 13(11):2709. doi: 10.3390/cancers13112709 34070941 PMC8199274

[35] Zhu H Li Q Zhao Y Peng H Guo L Zhu J. Vaccinia-Related Kinase 2 Drives Pancreatic Cancer Progression by Protecting Plk1 From Chfr-Mediated Degradation. Oncogene (2021) 40(28):4663–74. doi: 10.1038/s41388-021-01893-4 34140642

[36] Mok WC Wasser S Tan T Lim SG. Polo-Like Kinase 1, a New Therapeutic Target in Hepatocellular Carcinoma. World J Gastroenterol (2012) 18(27):3527–36. doi: 10.3748/wjg.v18.i27.3527 PMC3400854 22826617

[37] Dimri M Satyanarayana A. Molecular Signaling Pathways and Therapeutic Targets in Hepatocellular Carcinoma. Cancers (Basel) (2020) 12(2):491. doi: 10.3390/cancers12020491 PMC7072513 32093152

[38] Sheng H Huang Y Xiao Y Zhu Z Shen M Zhou P. ATR Inhibitor AZD6738 Enhances the Antitumor Activity of Radiotherapy and Immune Checkpoint Inhibitors by Potentiating the Tumor Immune Microenvironment in Hepatocellular Carcinoma. J Immunother Cancer (2020) 8(1):e000340. doi: 10.1136/jitc-2019-000340 32461345 PMC7254123

[39] Lei Y Wang S Liu J Yan W Han P Tian D. Identification of MCM Family as Potential Therapeutic and Prognostic Targets for Hepatocellular Carcinoma Based on Bioinformatics and Experiments. Life Sci (2021) 272:119227. doi: 10.1016/j.lfs.2021.119227 33607151

[40] Liu Z Mo H Liu R Niu Y Chen T Xu Q. Matrix Stiffness Modulates Hepatic Stellate Cell Activation Into Tumor-Promoting Myofibroblasts *via* E2F3-Dependent Signaling and Regulates Malignant Progression. Cell Death Dis (2021) 12(12):1134. doi: 10.1038/s41419-021-04418-9 34873170 PMC8648844

[41] Domingues P Gonzalez-Tablas M Otero A Pascual D Miranda D Ruiz L. Tumor Infiltrating Immune Cells in Gliomas and Meningiomas. Brain Behav Immun (2016) 53:1–15. doi: 10.1016/j.bbi.2015.07.019 26216710

[42] Sun H Liu L Huang Q Liu H Huang M Wang J. Accumulation of Tumor-Infiltrating CD49a(+) NK Cells Correlates With Poor Prognosis for Human Hepatocellular Carcinoma. Cancer Immunol Res (2019) 7(9):1535–46. doi: 10.1158/2326-6066.CIR-18-0757 31311791

[43] Stockinger B Veldhoen M. Differentiation and Function of Th17 T Cells. Curr Opin Immunol (2007) 19(3):281–6. doi: 10.1016/j.coi.2007.04.005 17433650

[44] Lee YK Turner H Maynard CL Oliver JR Chen D Elson CO. Late Developmental Plasticity in the T Helper 17 Lineage. Immunity (2009) 30(1):92–107. doi: 10.1016/j.immuni.2008.11.005 19119024 PMC3607320

[45] Bettelli E Carrier Y Gao W Korn T Strom TB Oukka M. Reciprocal Developmental Pathways for the Generation of Pathogenic Effector TH17 and Regulatory T Cells. Nature (2006) 441(7090):235–8. doi: 10.1038/nature04753 16648838

[46] Chatterjee S Daenthanasanmak A Chakraborty P Wyatt MW Dhar P Selvam SP. CD38-NAD(+)Axis Regulates Immunotherapeutic Anti-Tumor T Cell Response. Cell Metab (2018) 27(1):85–100.e8. doi: 10.1016/j.cmet.2017.10.006 29129787 PMC5837048

[47] Karpisheh V Ahmadi M Abbaszadeh-Goudarzi K Mohammadpour Saray M Barshidi A Mohammadi H. The Role of Th17 Cells in the Pathogenesis and Treatment of Breast Cancer. Cancer Cell Int (2022) 22(1):108. doi: 10.1186/s12935-022-02528-8 35248028 PMC8897940

[48] Wculek SK Cueto FJ Mujal AM Melero I Krummel MF Sancho D. Dendritic Cells in Cancer Immunology and Immunotherapy. Nat Rev Immunol (2020) 20(1):7–24. doi: 10.1038/s41577-019-0210-z 31467405

[49] Martinek J Wu TC Cadena D Banchereau J Palucka K. Interplay Between Dendritic Cells and Cancer Cells. Int Rev Cell Mol Biol (2019) 348:179–215. doi: 10.1016/bs.ircmb.2019.07.008 31810553

[50] Shaul ME Fridlender ZG. Neutrophils as Active Regulators of the Immune System in the Tumor Microenvironment. J Leukoc Biol (2017) 102(2):343–9. doi: 10.1189/jlb.5MR1216-508R 28264904

[51] Roybal KT Williams JZ Morsut L Rupp LJ Kolinko I Choe JH. Engineering T Cells With Customized Therapeutic Response Programs Using Synthetic Notch Receptors. Cell (2016) 167(2):419–32.e16. doi: 10.1016/j.cell.2016.09.011 27693353 PMC5072533

[52] Orecchioni M Bedognetti D Newman L Fuoco C Spada F Hendrickx W. Single-Cell Mass Cytometry and Transcriptome Profiling Reveal the Impact of Graphene on Human Immune Cells. Nat Commun (2017) 8(1):1109. doi: 10.1038/s41467-017-01015-3 29061960 PMC5653675

[53] Mantovani A Romero P Palucka AK Marincola FM. Tumour Immunity: Effector Response to Tumour and Role of the Microenvironment. Lancet (2008) 371(9614):771–83. doi: 10.1016/S0140-6736(08)60241-X 18275997

[54] Tonetti CR de Souza-Araujo CN Yoshida A da Silva RF Alves PCM Mazzola TN. Ovarian Cancer-Associated Ascites Have High Proportions of Cytokine-Responsive CD56bright NK Cells. Cells (2021) 10(7):1702. doi: 10.3390/cells10071702 34359872 PMC8306021

[55] Nie Y Liu D Yang W Li Y Zhang L Cheng X. Increased Expression of TIGIT and KLRG1 Correlates With Impaired CD56(bright) NK Cell Immunity in HPV16-Related Cervical Intraepithelial Neoplasia. Virol J (2022) 19(1):68. doi: 10.1186/s12985-022-01776-4 35413989 PMC9003970

[56] Duan J Lv G Zhu N Chen X Shao Y Liu Y. Multidimensional Profiling Depicts Infiltrating Immune Cell Heterogeneity in the Tumor Microenvironment of Stage IA Non-Small Cell Lung Cancer. Thorac Cancer (2022) 13(7):947–55. doi: 10.1111/1759-7714.14329 PMC8977165 35150094

[57] Chen Z Wang N Yao Y Yu D. Context-Dependent Regulation of Follicular Helper T Cell Survival. Trends Immunol (2022) 43(4):309–21. doi: 10.1016/j.it.2022.02.002 35249831

[58] Walker LSK. The Link Between Circulating Follicular Helper T Cells and Autoimmunity. Nat Rev Immunol (2022) 11:1–9. doi: 10.1038/s41577-022-00693-5 PMC8915145 35277664

[59] Galdiero MR Varricchi G Marone G. The Immune Network in Thyroid Cancer. Oncoimmunology (2016) 5(6):e1168556. doi: 10.1080/2162402X.2016.1168556 27471646 PMC4938375

[60] Bian J Lin J Long J Yang X Yang X Lu X. T Lymphocytes in Hepatocellular Carcinoma Immune Microenvironment: Insights Into Human Immunology and Immunotherapy. Am J Cancer Res (2020) 10(12):4585–606. PMC7783774 33415021

[61] Chen H Gao S Liu W Wong CC Wu J Wu J. RNA N(6)-Methyladenosine Methyltransferase METTL3 Facilitates Colorectal Cancer by Activating the M(6)A-GLUT1-Mtorc1 Axis and Is a Therapeutic Target. Gastroenterology (2021) 160(4):1284–300.e16. doi: 10.1053/j.gastro.2020.11.013 33217448

[62] Zhang J Hu K Yang YQ Wang Y Zheng YF Jin Y. LIN28B-AS1-IGF2BP1 Binding Promotes Hepatocellular Carcinoma Cell Progression. Cell Death Dis (2020) 11(9):741. doi: 10.1038/s41419-020-02967-z 32917856 PMC7486890

[63] Jiang W Cheng X Wang T Song X Zheng Y Wang L. LINC00467 Promotes Cell Proliferation and Metastasis by Binding With IGF2BP3 to Enhance the mRNA Stability of TRAF5 in Hepatocellular Carcinoma. J Gene Med (2020) 22(3):e3134. doi: 10.1002/jgm.3134 31656043

[64] Su T Huang M Liao J Lin S Yu P Yang J. Insufficient Radiofrequency Ablation Promotes Hepatocellular Carcinoma Metastasis Through N6-Methyladenosine mRNA Methylation-Dependent Mechanism. Hepatology (2021) 74(3):1339–56. doi: 10.1002/hep.31766 33638162

[65] Song Y He S Ma X Zhang M Zhuang J Wang G. RBMX Contributes to Hepatocellular Carcinoma Progression and Sorafenib Resistance by Specifically Binding and Stabilizing BLACAT1. Am J Cancer Res (2020) 10(11):3644–65. PMC7716158 33294259

[66] Cai X Chen Y Man D Yang B Feng X Zhang D. RBM15 Promotes Hepatocellular Carcinoma Progression by Regulating N6-Methyladenosine Modification of YES1 mRNA in an IGF2BP1-Dependent Manner. Cell Death Discov (2021) 7(1):315. doi: 10.1038/s41420-021-00703-w 34707107 PMC8551180

[67] Chen M Wei L Law CT Tsang FH Shen J Cheng CL. RNA N6-Methyladenosine Methyltransferase-Like 3 Promotes Liver Cancer Progression Through YTHDF2-Dependent Posttranscriptional Silencing of SOCS2. Hepatology (2018) 67(6):2254–70. doi: 10.1002/hep.29683 29171881

