# Supplementary figures and images for: Identification of CFHR4 as a Potential Prognosis Biomarker Associated With lmmune Infiltrates in Hepatocellular Carcinoma
Source: Front Immunol. 2022 Jun 22;13:892750. doi: 10.3389/fimmu.2022.892750 (PMC9257081; doi:10.3389/fimmu.2022.892750)

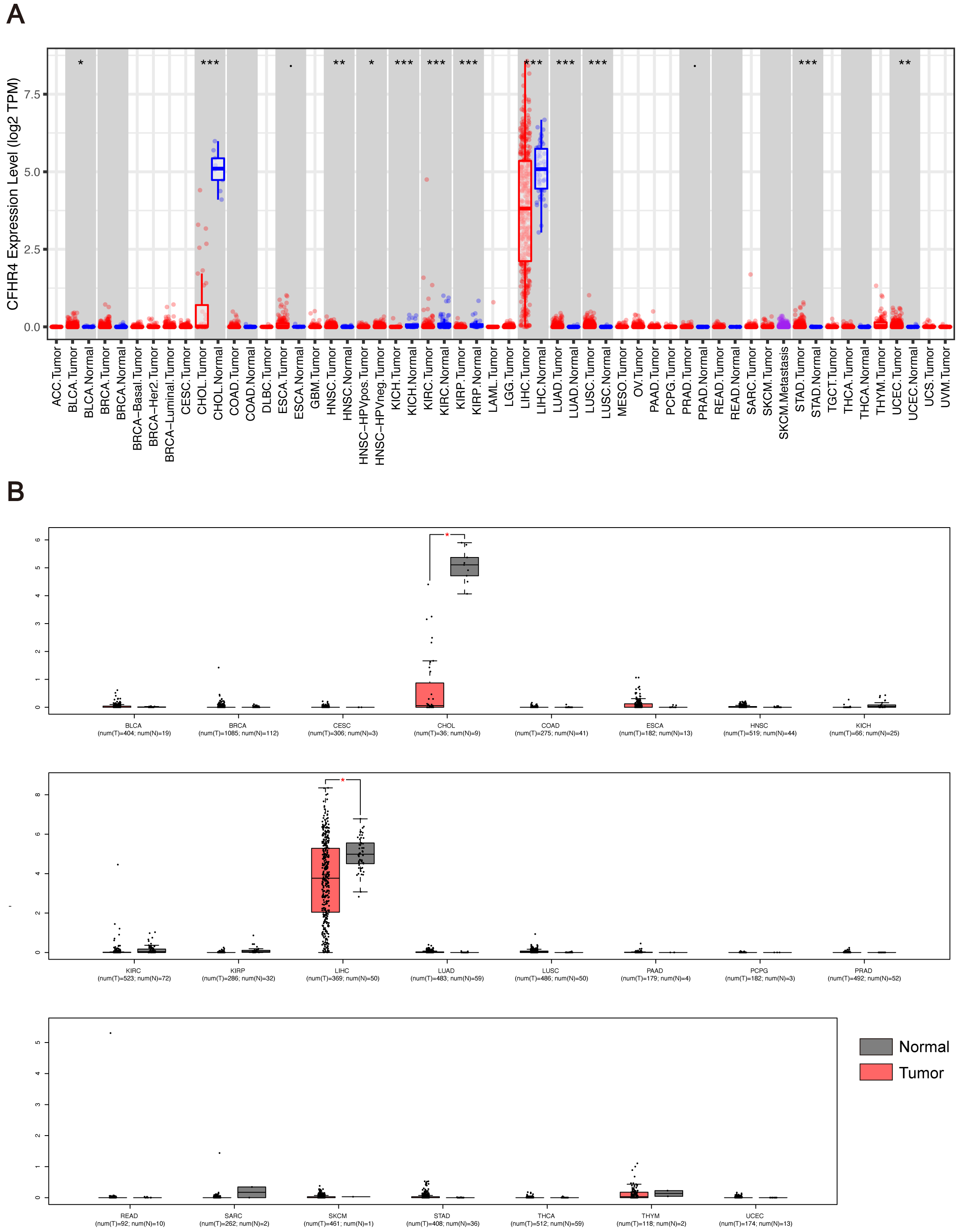

Supplement: Supplementary Figure 1 — CFHR4 expression levels in different cancer tissues compared to normal tissues. (A) CFHR4 expression levels in different cancer tissues compared to normal tissues in the Timer database. (B) CFHR4 expression levels in different cancer tissues compared to normal tissues in the GEPIA database. *p < 0.05, **p < 0.01, ***p < 0.001, NS, no significance. [file Image_1.tif]

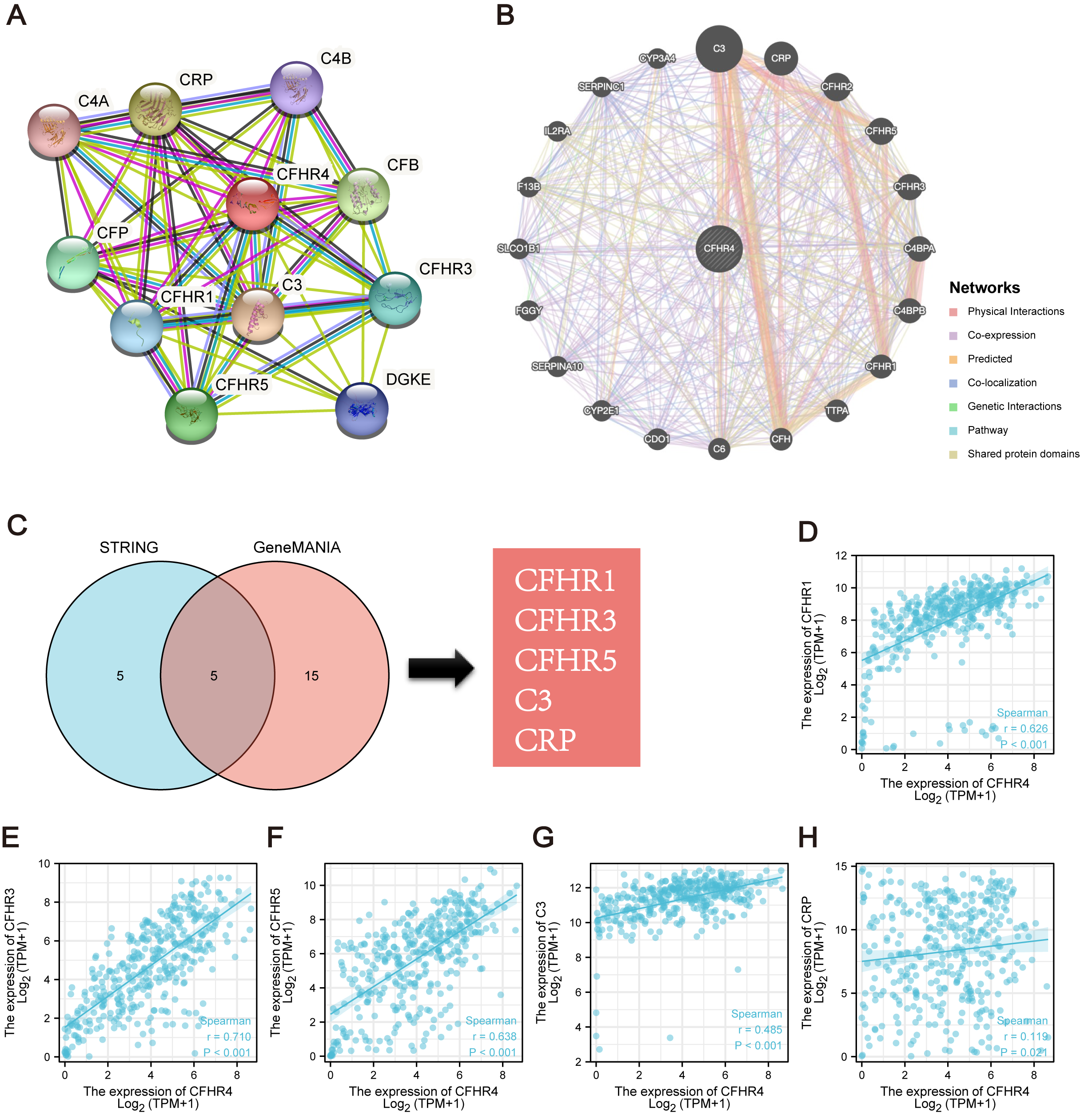

Supplement: Supplementary Figure 2 — PPI network and potential CFHR4-interacting target genes. (A) PPI networks were built using the STRING database. (B) PPI networks were built using the GeneMANIA database. (C) The intersecting genes identified by the STRING and GeneMANIA online databases are displayed in a Venn diagram. (D) Scatter plot showing the correlation between CFHR1 and CFHR4 expression. (E) Scatter plot showing the correlation between CFHR3 and CFHR4 expression. (F) Scatter plot showing the correlation between CFHR5 and CFHR4 expression. (G) Scatter plot showing the correlation between C3 and CFHR4 expression. (H) Scatter plot showing the correlation between CRP and CFHR4 expression. [file Image_2.tif]

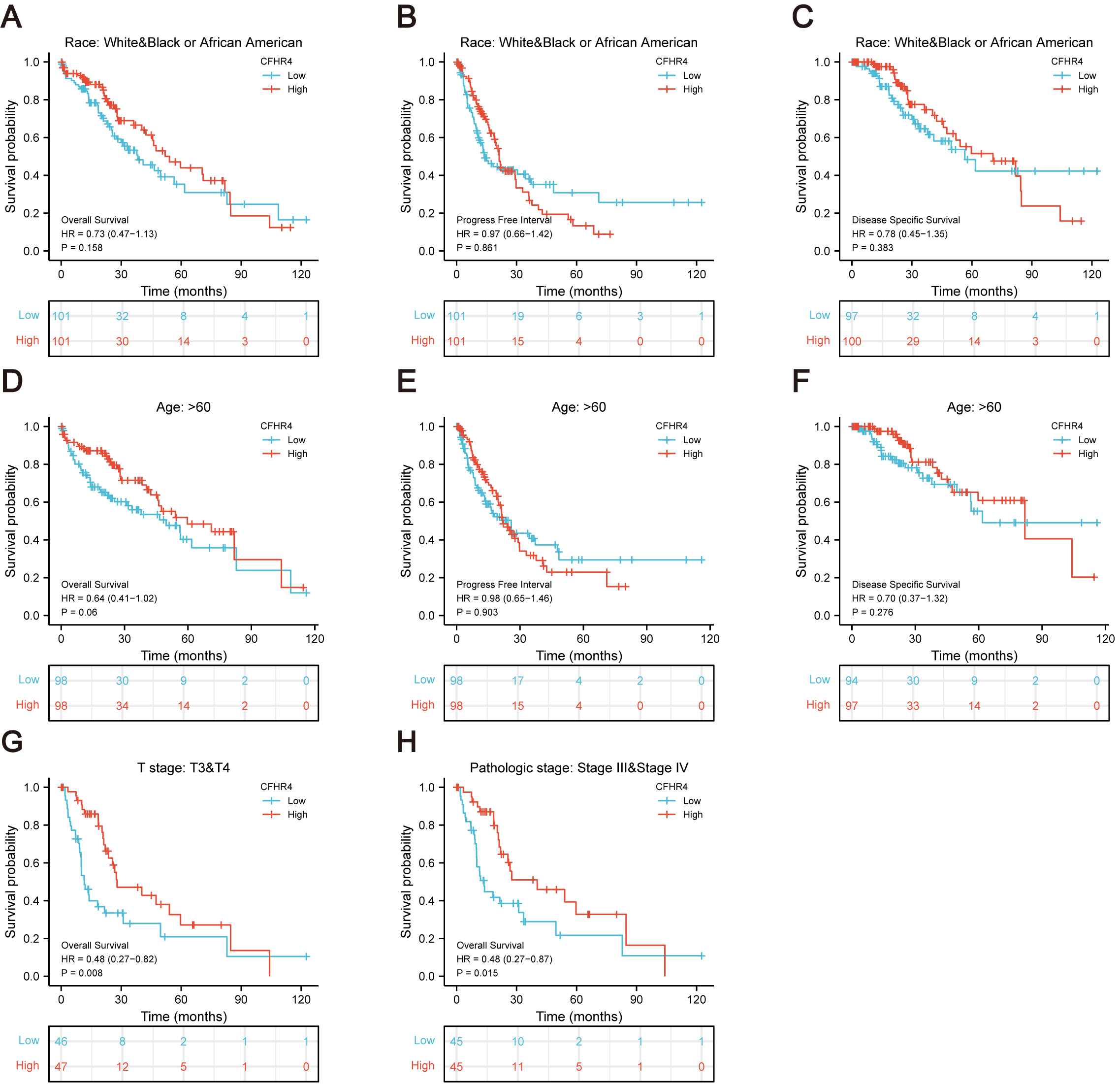

Supplement: Supplementary Figure 3 — The prognostic value of CFHR4 in HCC. (A–C) OS, DSS and PFI survival curves for white, black or African–American patients with HCC presenting high and low CFHR4 expression. (D-F) OS, DSS and PFI survival curves for patients with HCC aged > 60 years presenting with high and low CFHR4 expression. (G) OS curves for patients with stage T3 and T4 HCC presenting with high and low CFHR4 expression. (H) OS curves for patients with pathological stage III and IV HCC presenting with high and low CFHR4 expression. [file Image_3.tif]
