# Supplementary material for: Identification of CFHR4 as a Potential Prognosis Biomarker Associated With lmmune Infiltrates in Hepatocellular Carcinoma
Source: Front Immunol. 2022 Jun 22;13:892750. doi: 10.3389/fimmu.2022.892750 (PMC9257081; doi:10.3389/fimmu.2022.892750)
Supplement: Supplementary Table 6 — OS of patients with HCC based on prognostic covariates. [file Table_6.docx]

| **Characteristics** | **Total(N)** | **Univariate analysis** | |
| --- | --- | --- | --- |
|  |  | **Hazard ratio (95% CI)** | **P value** |
| T stage | 370 |  |  |
| T1 | 183 | Reference |  |
| T2 | 94 | 1.431 (0.902-2.268) | 0.128 |
| T3 | 80 | 2.674 (1.761-4.060) | <0.001 |
| T4 | 13 | 5.386 (2.690-10.784) | <0.001 |
| N stage | 258 |  |  |
| N0 | 254 | Reference |  |
| N1 | 4 | 2.029 (0.497-8.281) | 0.324 |
| M stage | 272 |  |  |
| M0 | 268 | Reference |  |
| M1 | 4 | 4.077 (1.281-12.973) | 0.017 |
| Pathologic stage | 349 |  |  |
| Stage I | 173 | Reference |  |
| Stage II&Stage III&Stage IV | 176 | 2.090 (1.429-3.055) | <0.001 |
| Tumor status | 354 |  |  |
| Tumor free | 202 | Reference |  |
| With tumor | 152 | 2.317 (1.590-3.376) | <0.001 |
