# Supplementary material for: Identification of CFHR4 as a Potential Prognosis Biomarker Associated With lmmune Infiltrates in Hepatocellular Carcinoma
Source: Front Immunol. 2022 Jun 22;13:892750. doi: 10.3389/fimmu.2022.892750 (PMC9257081; doi:10.3389/fimmu.2022.892750)
Supplement: Supplementary Table 7 — DSS patients with HCC based on prognostic covariates. [file Table_7.docx]

| **Characteristics** | **Total(N)** | **Univariate analysis** | |
| --- | --- | --- | --- |
|  |  | **Hazard ratio (95% CI)** | **P value** |
| T stage | 362 |  |  |
| T1 | 180 | Reference |  |
| T2 | 92 | 1.625 (0.872-3.028) | 0.126 |
| T3 | 77 | 3.748 (2.177-6.451) | <0.001 |
| T4 | 13 | 10.164 (4.480-23.060) | <0.001 |
| N stage | 253 |  |  |
| N0 | 249 | Reference |  |
| N1 | 4 | 3.612 (0.870-14.991) | 0.077 |
| M stage | 268 |  |  |
| M0 | 265 | Reference |  |
| M1 | 3 | 5.166 (1.246-21.430) | 0.024 |
| Pathologic stage | 341 |  |  |
| Stage I | 170 | Reference |  |
| Stage II&Stage III&Stage IV | 171 | 2.909 (1.718-4.925) | <0.001 |
| Tumor status | 354 |  |  |
| Tumor free | 202 | Reference |  |
| With tumor | 152 | 775790759.389 (0.000-Inf) | 0.994 |
