# Supplementary material for: Identification of CFHR4 as a Potential Prognosis Biomarker Associated With lmmune Infiltrates in Hepatocellular Carcinoma
Source: Front Immunol. 2022 Jun 22;13:892750. doi: 10.3389/fimmu.2022.892750 (PMC9257081; doi:10.3389/fimmu.2022.892750)
Supplement: Supplementary Table 8 — PFI of patients with HCC based on prognostic covariates. [file Table_8.docx]

| **Characteristics** | **Total(N)** | **Univariate analysis** | |
| --- | --- | --- | --- |
|  |  | **Hazard ratio (95% CI)** | **P value** |
| T stage | 370 |  |  |
| T1 | 183 | Reference |  |
| T2 | 94 | 2.020 (1.411-2.892) | <0.001 |
| T3 | 80 | 2.620 (1.811-3.789) | <0.001 |
| T4 | 13 | 4.266 (2.181-8.347) | <0.001 |
| N stage | 258 |  |  |
| N0 | 254 | Reference |  |
| N1 | 4 | 1.370 (0.338-5.552) | 0.659 |
| M stage | 272 |  |  |
| M0 | 268 | Reference |  |
| M1 | 4 | 3.476 (1.091-11.076) | 0.035 |
| Pathologic stage | 349 |  |  |
| Stage I | 173 | Reference |  |
| Stage II&Stage III&Stage IV | 176 | 2.284 (1.670-3.122) | <0.001 |
| Tumor status | 354 |  |  |
| Tumor free | 202 | Reference |  |
| With tumor | 152 | 11.342 (7.567-17.000) | <0.001 |
